# Molecular forecasting of domoic acid during a pervasive toxic diatom bloom

**DOI:** 10.1073/pnas.2319177121

**Published:** 2024-09-19

**Authors:** John K. Brunson, Monica Thukral, John P. Ryan, Clarissa R. Anderson, Bethany C. Kolody, Chase C. James, Francisco P. Chavez, Chui Pin Leaw, Ariel J. Rabines, Pratap Venepally, Zoltan Fussy, Hong Zheng, Raphael M. Kudela, G. Jason Smith, Bradley S. Moore, Andrew E. Allen

**Affiliations:** ^a^Center for Marine Biotechnology and Biomedicine, Scripps Institution of Oceanography, University of California San Diego, La Jolla, CA 92093; ^b^Microbial and Environmental Genomics Group, J. Craig Venter Institute, La Jolla, CA 92037; ^c^Integrative Oceanography Division, Scripps Institution of Oceanography, University of California San Diego, La Jolla, CA 92093; ^d^Research Division, Monterey Bay Aquarium Research Institute, Moss Landing, CA 95093; ^e^Southern California Coastal Ocean Observing System, Scripps Institution of Oceanography, University of California San Diego, La Jolla, CA 92093; ^f^Innovative Genomics Institute, University of California, Berkeley, CA 94720; ^g^Department of Biological Sciences, University of Southern California, Los Angeles, CA 90089; ^h^Bachok Marine Research Station, Institute of Ocean and Earth Sciences, University of Malaya, Bachok, Kelantan 16310, Malaysia; ^i^Ocean Sciences Department, Institute of Marine Sciences, University of California-Santa Cruz, Santa Cruz, CA 95064; ^j^Environmental Biotechnology Department, Moss Landing Marine Laboratories, Moss Landing, CA 95039; ^k^Skaggs School of Pharmacy and Pharmaceutical Sciences, University of California San Diego, La Jolla, CA 92093

**Keywords:** harmful algal bloom, nutrient stress, global change, domoic acid biosynthesis, diatom

## Abstract

In 2015, the largest recorded harmful algal bloom (HAB) occurred in the Northeast Pacific, causing nearly 100 million dollars in damages to fisheries and killing many protected marine mammals. Dominated by the toxic diatom *Pseudo-nitzschia australis*, this bloom produced high levels of the neurotoxin domoic acid (DA). Through molecular and transcriptional characterization of 52 near-weekly phytoplankton net-tow samples collected at a bloom hotspot in Monterey Bay, California, we identified active transcription of known DA biosynthesis (*dab*) genes from the three identified toxigenic species, including *P. australis* as the primary origin of toxicity. Elevated expression of silicon transporters (*sit1*) during the bloom supports the previously hypothesized role of dissolved silica (Si) exhaustion in contributing to bloom physiology and toxicity. We find that coexpression of the *dabA* and *sit1* genes serves as a robust predictor of DA one week in advance, potentially enabling the forecasting of DA-producing HABs. We additionally present evidence that low levels of iron could have colimited the diatom population along with low Si. Iron limitation represents an overlooked driver of both toxin production and ecological success of the low-iron-adapted *Pseudo-nitzschia* genus during the 2015 bloom, and increasing pervasiveness of iron limitation may fuel the escalating magnitude and frequency of toxic *Pseudo-nitzschia* blooms globally. Our results advance understanding of bloom physiology underlying toxin production, bloom prediction, and the impact of global change on toxic blooms.

## Significance

*Pseudo-nitzschia* diatoms form oceanic harmful algal blooms that threaten human health through production of the neurotoxin domoic acid (DA). DA biosynthetic gene expression is hypothesized to control DA production in the environment, yet what regulates expression of these genes is poorly understood. In this study, we uncovered expression of DA biosynthesis genes by multiple toxigenic *Pseudo-nitzschia* species during an economically impactful bloom along the North American West Coast and identified genes that predict DA in advance of its production. We found that iron and silica colimitation restrained the bloom and likely promoted toxin production. This work suggests that increasing iron limitation due to global change may play a previously unrecognized role in driving bloom frequency and toxicity.

Harmful algal blooms (HABs) produce a variety of potent molecular toxins, including many that pose an active threat to human health (1). Bioaccumulation of the diatom-produced neurotoxin domoic acid (DA) in various seafoods such as Dungeness crab and mussels routinely triggers fishery closures around the world, resulting in millions of dollars of economic losses during toxic events (2). Acute human exposure to DA can lead to amnesic shellfish poisoning (ASP), a potentially lethal syndrome characterized by seizures, vomiting, and short-term memory loss (3, 4). Because of the risks to human health afforded by DA and other algal toxins, routine environmental monitoring is implemented to better predict and respond to major HAB events (5–7).

Blooms of the DA-producing diatom genus *Pseudo-nitzschia* are near-annual events along the North American West Coast and are fueled by strong seasonal upwelling in the California Current Ecosystem (CCE) (8, 9). In 2015, a bloom of *Pseudo-nitzschia australis* (*P. australis*) extended from the Gulf of Alaska to Point Conception, California from spring to late summer and, to date, is the largest and longest recorded HAB in the Northeast Pacific Ocean. Toxic populations of *P. australis*, a species common to the central California coast, expanded their range northward in association with an extreme marine heatwave in 2014 to 2015 (10, 11). During this historically large HAB event, exceptionally high levels of DA were detected in Monterey Bay, California, prompting further study into the oceanographic mechanisms fueling the bloom event in the bay (12, 13). Monterey Bay experiences frequent phytoplankton blooms driven by strong seasonal upwelling at Point Año Nuevo to the north and Point Sur to the south (*SI Appendix*, Fig. S1) (14–16). Such upwelling waters, with elevated concentrations of dissolved nitrogen and phosphorus, can often drive phytoplankton communities into Si and/or iron limitation (17–19). Iron limitation has been shown to limit growth of coastal planktonic communities, and iron stress has been shown to up-regulate DA production (20–22). The highly toxic *P. australis* HAB of 2015 coincided with historically low Si concentrations recorded throughout Monterey Bay, and limitation of this key nutrient has been shown to induce DA production in laboratory culture and modeling experiments (12, 23–27). However, a wide variety of biotic and abiotic factors have been shown to influence toxin production by *Pseudo-nitzschia*. Despite decades of research on DA production in the lab and in mesoscale oceanographic studies, a predictable set of physiological mechanisms underpinning DA production in the environment remains unclear.

The DA biosynthetic pathway in *Pseudo-nitzschia multiseries* was first discovered by using an RNA-sequencing approach to identify DA biosynthetic (*dab)* genes that were up-regulated under simultaneous phosphate limitation and elevated *p*CO_2_, conditions that increase DA production in laboratory cultures (28, 29). The enzymes encoded by *dabA*, *dabC,* and *dabD* were demonstrated to perform key biosynthetic reactions required for in vitro production of the DA molecule (28, 30). Among diatoms, *dab* genes have only been described within the genus *Pseudo-nitzschia* in the species *P. multiseries*, *P. multistriata*, *P. australis*, and *P. seriata* (28, 31). Following this initial discovery, homologues to the *dab* genes have also been characterized in red algae that produce either DA or the structurally related neurochemical kainic acid (32, 33).

Molecular methodology, targeting DNA and/or RNA, can enable rapid identification of HAB species at significantly improved precision and has been successfully deployed for routine monitoring of *Pseudo-nitzschia* in Monterey Bay (34). Additionally, detection of toxin biosynthetic genes has been proposed as a possible route for monitoring and forecasting HAB toxicity, and the cyanobacterial HAB community has implemented molecular monitoring guided by the biosynthetic genes for toxins such as microcystin and saxitoxin (35, 36). Environmental detection of *dab* transcripts is currently not implemented in the monitoring of DA-producing *Pseudo-nitzschia* HABs, and at present it is unknown how *dab* transcription might be related to DA production in the marine environment.

**Fig. 1.**
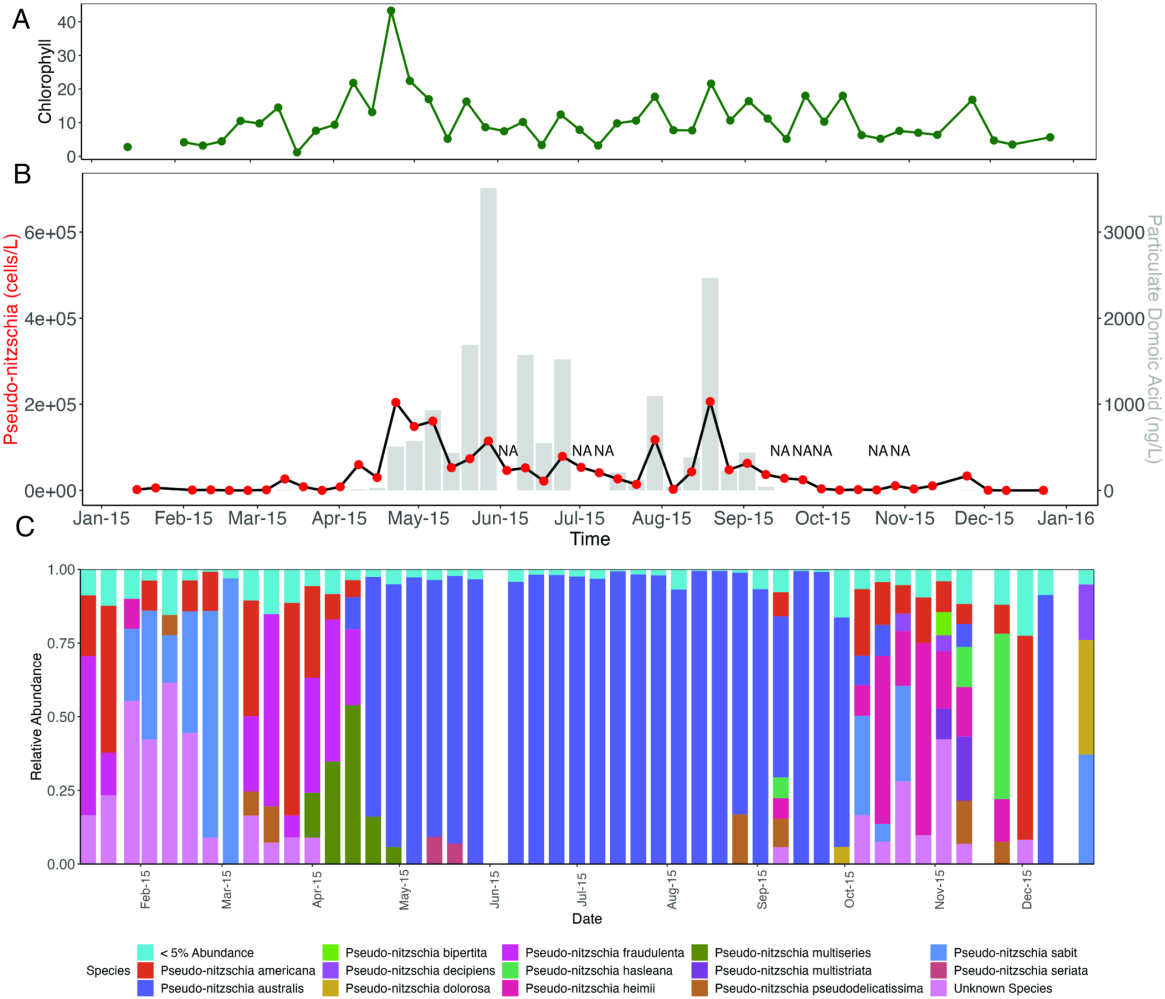
*Pseudo-nitzschia* targeted ITS2 amplicon sequencing. (*A*) Chlorophyll*-a* (mg/m^3^) at MWII, (*B*) *Pseudo-nitzschia* cell counts from weekly MWII phytoplankton net tows (red dots, *Left* axis) overlayed with particulate domoic acid (pDA, ng/L) measurements from filtered seawater (gray bars, *Right* axis), as reported by CalHABMAP monitoring^7^ and (*C*) Relative abundance of *Pseudo-nitzschia* species as determined by ITS2 amplicon sequencing. Non-ITS2 sequences were removed prior to analysis. NA indicates missing data.

In this study, we targeted rRNA and mRNA to combine 18S and ITS2 metabarcoding with polyA-enriched RNA-sequencing to provide a framework for characterizing toxic *Pseudo-nitzschia* species, *dab*, and other gene transcription at the molecular level. These metabarcoding and metatranscriptomic resources were generated from 52 near-weekly phytoplankton net-tow samples collected at Monterey Municipal Wharf II (MWII) in Monterey Bay, California from late 2014 to the end of 2015, encapsulating the entire *P. australis* bloom event (Fig. 1 and *SI Appendix*, Table S1 and Zenodo https://zenodo.org/records/10728894). Analysis of molecular barcoding datasets revealed the phytoplankton succession as community composition transitioned from nontoxic diatom species to a near-monospecific bloom of DA-producing *Pseudo-nitzschia australis*. We also measured transcription of *dab* genes in the metatranscriptomics dataset and compared these findings with particulate DA (pDA, nanograms per liter of filtered seawater) measurements taken at MWII and abundances of toxigenic species as revealed by metabarcoding. We focused on how expression patterns of *dab* genes and DA production related to a silicon transporter (*sit1*), a carbonate-dependent phytotransferrin, iron starvation–induced protein (*ISIP2A*), and ferritin (*FTN*). In addition, the metatranscriptomics data allowed us to further investigate highly expressed transcripts to describe the shifting physiology of the *P. australis* bloom throughout the entire HAB event.

## Results

### Amplicon Sequencing Reveals the Spring Phytoplankton Succession.

Temperature and nitrate measurements at MWII in 2015 suggested that cold, nutrient-rich upwelling began in late March and early April, supported by geophysical and ecosystem dynamics studies (*SI Appendix*, Fig. S2) (12). Amplicon sequencing of the 18S rRNA-V4 region revealed a stark transition from a zooplankton-dominant ecosystem characterized by abundant dinoflagellates (Myzozoa) to a diatom-dominant (Bacillariophyta) community in early to mid-March, a shift coincident with increased chlorophyll concentrations at MWII (*SI Appendix*, Fig. S3*A*, Fig. 1*A*). Diatoms remained the dominant members of the microalgal community by 18S amplicons until mid-July when their relative abundance briefly decreased. The diatom-dominated phytoplankton bloom returned once more in August before its ultimate demise at the end of September, giving way to an intermittent assemblage of diatoms and dinoflagellates, copepods, and other grazing zooplankton in the Autumn months.

The early spring bloom was dominated by centric diatoms, primarily *Chaetoceros*, prior to transitioning in late April to a community composed almost entirely of *Pseudo-nitzschia australis* that dominated the rest of the bloom season through early autumn (*SI Appendix*, Figs. S3*B*, S4, and S5). Such phytoplankton succession from a mixed assemblage community during new upwelling to a diatom-dominated community postupwelling has been previously reported in the CCE (37, 38). These observations are further supported by chloroplast sequences recovered from 16S rRNA sequencing (*SI Appendix*, Fig. S6). Returning diatom assemblages present in October and November were, like the early spring phytoplankton community, primarily composed of *Chaetoceros* (*SI Appendix*, Fig. S6). Alpha-diversity analysis of the 18S-V4 data revealed that diatom diversity indeed declined during the bloom event, concurrent with previous observations suggesting a near-monospecific bloom of *P. australis*. This significant reduction in diversity was primarily driven by pennate diatoms resulting from *Pseudo-nitzschia* dominance, with centric diatom diversity appearing largely unaffected throughout the *P. australis* bloom (*SI Appendix*, Fig. S7 *A* and *B*).

### ITS2 Sequencing Reveals *Pseudo-nitzschia* Species Diversity Prior to *P. australis* Dominance.

While the Spring phytoplankton succession was resolved to the genus level by 18S rRNA metabarcoding, amplicon sequencing of the internal transcribed spacer 2 (ITS2) region was implemented to improve characterization of *Pseudo-nitzschia* species succession. The ITS2 region has been demonstrated to provide species-level resolution consistent with classical methods of species delineation, such as electron microscopy (39). ITS2 metabarcoding confirmed *P. australis* as the dominant species during the bloom event (10, 13). The early spring phytoplankton succession dominated by *Chaetoceros* appeared to also include low levels of various *Pseudo-nitzschia* species such as *P. fraudulenta, P. cuspidata*, *P. americana*, and *P. pungens*. All these species, barring *P. americana*, have been demonstrated to produce low-to-modest amounts of DA in the lab, and their presence here coincides with very low levels of particulate DA detected at MWII (~7 ng/L pDA, March 11th, Fig. 1) (40, 41).

Beginning in late April, the community shifted to near-complete dominance of *P. australis*, the species previously identified to be the main driver of the bloom in Monterey Bay and along the entire North American West Coast (10, 12, 13). In addition, a substantial subpopulation of potentially toxic *P. multiseries* was also identified throughout the month of April in conjunction with modestly increased, but still low, pDA concentrations (~22 ng/L, April 15th, Fig. 1). Detection of the *P. multiseries* subpopulation at MWII is corroborated by *P. multiseries* presence during a similar period at the northern point of Monterey Bay at Santa Cruz Wharf (13). A third subpopulation of the potentially highly toxigenic *P. seriata* was also observed in early to mid-May, albeit at a much lower level of abundance than *P. australis*. These data suggest that several species may have contributed to pDA production throughout different phases of the bloom season and that a sizable population of *P. multiseries* may have been a significant contributor to initial pDA production at MWII prior to the emergence of *P. australis* as the dominant species.

### Expression of *dab* Genes Reflects Toxin Production by *Pseudo-nitzschia*.

We reconstructed the sequence and expression profiles of several *dab* transcripts in the polyA-enriched RNA-sequencing dataset. These transcripts displayed nearly identical translated amino acid sequences compared to known *dab* genes from *P. australis, P. multiseries*, and *P. seriata*, three species identified by ITS2 sequencing during the bloom (*SI Appendix*, Table S2) (28, 31). The full suite of core DA biosynthetic genes (*dabA*, *dabC*, and *dabD*) as expressed by the dominant bloom species, *P. australis*, was identified and was detected throughout the entire bloom event (April 15th to September 30th). Assemblies also yielded *P. multiseries* transcripts for *dabA* and *dabC*, both of which displayed expression profiles coinciding with the detection of the species through ITS2 amplicon sequencing (April 1st to mid-May). Two additional *dab* genes were identified from the de novo assembly: One could be confidently assigned to *P. seriata* (*dabA*, April 22nd to June 10th), and the other is of unknown origin (*dabC*, March 11th to April 8th). The unknown *dabC* transcript was present early in the spring phytoplankton succession, coincident with the presence of various low-toxicity *Pseudo-nitzschia* spp. and low-level detection of particulate domoic acid (pDA). Active *dabA* and *dabC* gene transcription co-occurred with detection of pDA throughout most of the spring and summer months (*SI Appendix*, Table S3 and Fig. S8).

Phytoplankton sampling and pDA measurements taken from MWII, combined with molecular barcoding and *dab* transcription measurements, suggested two phases to the persistent *P. australis* bloom as observed from MWII. The first phase involved proliferation of the bloom and steady build-up of cellular DA (cDA), or pDA normalized per *Pseudo-nitzschia* cell, throughout April and May in conjunction with *dab* gene expression by the *P. australis* and *P. multiseries* subpopulations during these months (*SI Appendix*, Fig. S8*A*) (42). The second phase of the bloom occurred after the July disappearance of *Pseudo-nitzschia* and subsequent decrease in cDA (Fig. 1 and *SI Appendix*, Fig. S8*B*). Beginning in late July with the sudden resurgence of *Pseudo-nitzschia* cells and DA, periodic increases in cDA were accompanied by increases in the abundance of *P. australis dabA* and *dabC* transcripts during the late phase of the *Pseudo-nitzschia* HAB (Fig. 1*B* and *SI Appendix*, Fig. S8 *B*–*D*). In both early and late phases of the bloom, increases in cDA concentrations coincided with spikes in transcript abundance for *dabA* and *dabC*, the two potentially diagnostic DA biosynthetic genes (*SI Appendix*, Fig. S8 *B*–*D*) (28).

### Expression Profiles of *P. australis* Transcripts Reveal Evolution of the Bloom.

**Fig. 2.**
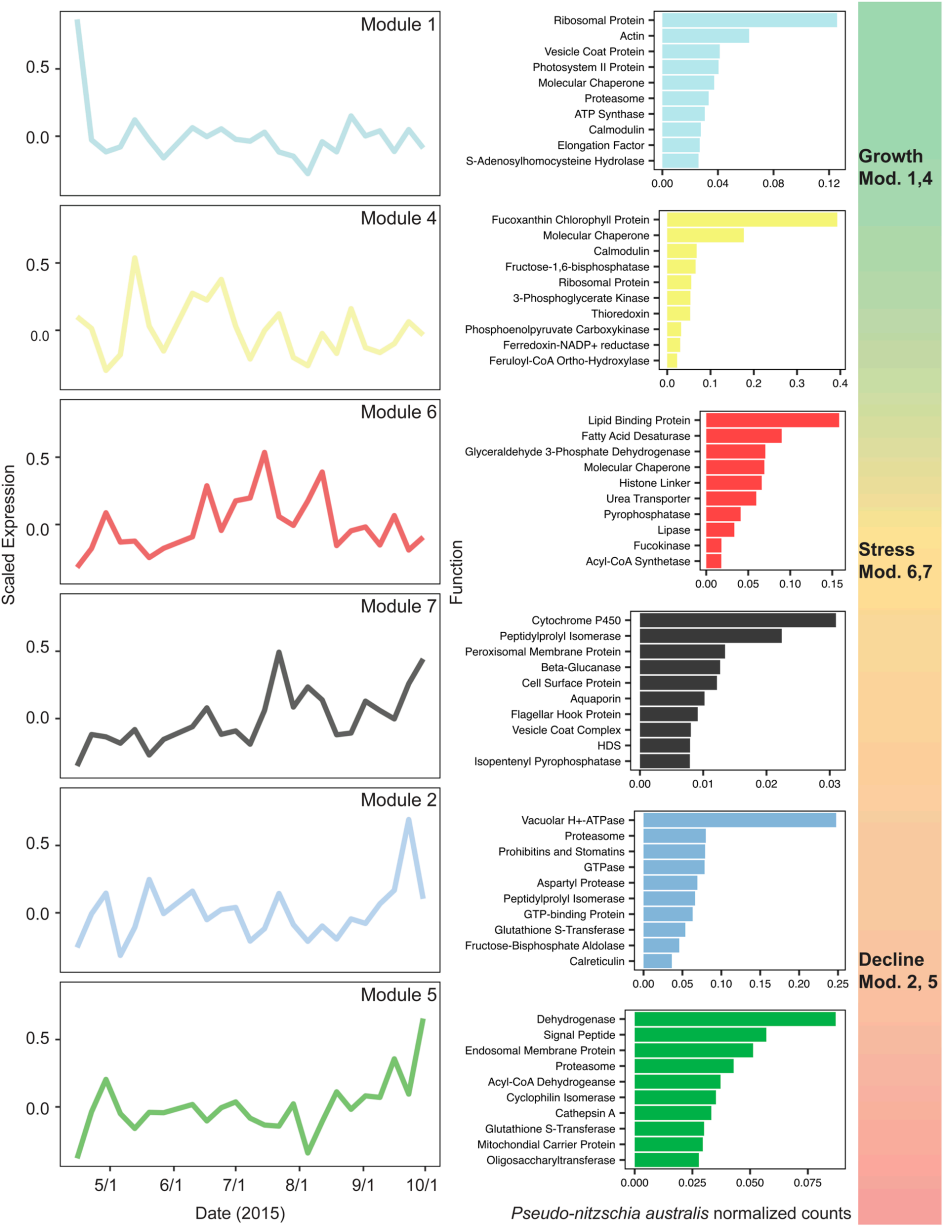
Patterns in coexpressed *P. australis* genes reveal bloom progression from growth to stress to decline during the duration of the HAB (April 15th to September 30th). The data represent relative expression modules for *P. australis* transcripts throughout the HAB as determined by WGCNA. Corresponding bar charts on the right of each module show the ten most abundant functions of open reading frames (ORFs) of each module using KEGG descriptions. To highlight dynamics associated with *Pseudo-nitzschia australis* transcriptome allocation remodeling, the data are shown as the sum of *Pseudo-nitzschia australis* normalized read counts. ORFs without annotations are not shown.

Weighted gene correlation network analysis (WGCNA) on a highly expressed subset of the de novo assembled *P. australis* HAB metatranscriptome from the bloom period (April 15th to September 30th) identified seven modules of transcripts with similar expression profiles (Fig. 2 and *SI Appendix*, Fig. S9) (43). Both *dabA* and *dabD* were found in module 7, along with two genes encoding key isoprenoid biosynthesis enzymes: 4-hydroxy-3-methylbut-2-enyl-diphosphate synthase (HDS) and isopentenyl-diphosphate delta-isomerase (IDI) (*SI Appendix*, Fig. S10*A*). Both proteins are directly implicated in biosynthesis of the DA precursor geranyl pyrophosphate and are known to be coexpressed with the *dab* gene products (31). Two additional isoprenoid biosynthesis transcripts, 1-deoxy-D-xylulose-5-phosphate synthase (DXS) and 4-hydroxy-3-methylbut-2-enyl diphosphate reductase (HDR), were found in modules 2 and 4, respectively. All four isoprenoid biosynthesis genes included in this analysis are implicated in the chloroplastic, nonmevalonate pathway thought to feed directly into DA biosynthesis (28, 31, 44).

The remaining DA biosynthetic gene *dabC* was found in module 4, together with HDR but separate from *dabA* and *dabD* (*SI Appendix*, Fig. S10*A*). While *dabC* is coexpressed with *dabA* and *dabD* in *P. multiseries* and *P. seriata* cultures, its enzyme DabC catalyzes the third biosynthetic reaction to DA in a suspected different cellular compartment than the DabA and DabD enzymes and thus might be differentially regulated (28, 31). Module 4 also displayed functional clustering of photosynthetic machinery like chlorophyll-binding proteins, light-harvesting complexes, the iron–sulfur subunit of the cytochrome b6f complex, photosystem I reaction center subunit VIII, photosystem II stabilizing-protein PsbU, ferredoxin-NADP^+^ reductase, transketolase, and fructose-1,6-bisphosphatase (45) (*SI Appendix*, Fig. S10*B*).

Module 1 includes transcripts that were abundant at the beginning of the bloom and are among the first *P. australis* transcripts detectable. Many of these transcripts are related to active cell growth and proliferation. Half of the 16 ribosomal subunit transcripts in the WGCNA dataset were found in module 1 (*SI Appendix*, Fig. S10*B*). Transcripts for F-type ATP synthase subunits were also primarily found in module 1, suggesting increased ATP production in conjunction with a contig encoding a mitochondrial ATP/ADP carrier protein, also found in the module. One V-ATPase subunit was found in this module along with clathrin, two proteins that play an important role in diatom cell division and cell wall silicification (46, 47). However, most of the V-type ATPase subunits in the analysis were found in module 2, perhaps implying dynamic regulation of V-type ATPase subunits for different cellular functions throughout different phases of the bloom.

Modules 2 and 5 include transcripts with higher relative abundance at the end of the bloom season, including some transcripts that may be linked to general physiological stress or cell death. Subunits of the proteasome, required for protein degradation and turnover, are enriched in these two modules. Out of 13 proteasome 20S/26S subunits in the WGCNA dataset, eight were found in module 2 or 5 (*SI Appendix*, Fig. S10*B*). Increased protein turnover versus new protein translation has been described previously in diatom systems as a side effect of decreased growth, cell division, and proliferation and may be associated with nutrient and temperature stress (48). In general, transcripts associated with protein turnover are enriched in these two modules, with 26 of 46 transcripts of relevant KOG class annotation (“Posttranslational modification, Protein turnover, Chaperones”) found in modules 2 and 5 (*SI Appendix*, Fig. S10*B*). Additional transcripts from modules 2 and 5 associated with protein turnover and related stress response include ubiquitin ligases, aspartyl protease, glutaredoxin and thioredoxin, cyclophilin, and chaperones DnaJ, GroES, and GroEL.

Module 6 includes select transcripts involved in diatom response to nitrogen starvation. Urea transporters and specific ammonia transporters are known to be up-regulated in diatoms under nitrogen-limiting conditions (49). Both a urea transporter and the *P. australis* homolog of AMT1_1, an ammonia transporter from *P. tricornutum* induced in low-N conditions, can be found in this module. Nitrogen limitation in diatoms is also coupled to an increase in fatty acid biosynthesis and accumulation. Three fatty acid desaturases, including a D9-desaturase up-regulated in *P. tricornutum* lacking functional nitrate reductase, were also observed in this module (50). Additional genetic markers of diatom physiological state, such as the Si starvation–induced silicon transporter *sit1* and the iron-responsive ferritin (FTN) and iron starvation–induced proteins (ISIPs), did not meet statistical cutoffs to be included in WGCNA, primarily due to their lack of transcription in a majority of the studied timepoints. This observation is likely due to intermittent induction of these transcripts in direct response to nutrient starvation–induced conditions.

### Episodic Summer Upwelling Perpetuated the Lengthy Bloom.

The decline in *Pseudo-nitzschia* cell counts in early July (Fig. 1*B*) coincided with rapid warming and freshening of the mixed layer in Monterey Bay (12). The strong freshening indicated influx of lower-salinity water from the inner California Current, a typical oceanographic response to relaxation of upwelling favorable winds (12, 16). Resurgence of the bloom at MWII in late July likely resulted from resumed upwelling that introduced nutrients to the bay. Following weak upwelling during the first half of July, episodic intensification of upwelling-favorable winds began in late July and persisted for months (12). The resumed influence of upwelling on the bay is evident in satellite SST images. In contrast to warm conditions observed within the bay and at the Point Año Nuevo upwelling center on July 20, cool upwelling plumes originating at Point Año Nuevo were observed to extend into Monterey Bay on July 27 and August 14 (*SI Appendix*, Fig. S11). Amplicon sequencing revealed a planktonic ecosystem trending toward a grazer and dinoflagellate-dominated population following the late June freshening event from July 1st through July 15th, which gave way to a majority population of copepod grazers on July 22nd (*SI Appendix*, Fig. S3*A*). On July 29th, a substantial population of diatoms emerged that disappeared from the dataset for just one week, on August 5th. Starting August 12th, diatoms fully returned to MWII and remained until September 30th (*SI Appendix*, Fig. S3*A*). Among diatoms, *Chaetoceros spp.* represented the largest fraction of the population on July 22nd and July 29th before they gave way to *Pseudo-nitzschia* for the full month of August (*SI Appendix*, Fig. S6).

### Multiple Lines of Evidence Suggest that the *P. australis* Bloom was Limited by Silica and Iron.

During the relaxation of upwelling in early July, silica concentrations in the water column fell to a historic low and dropped below nitrate concentrations, indicative of silica starvation (12) (*SI Appendix*, Fig. S12). Silica limitation decreases diatom growth rates and, under starvation conditions, can arrest the cell cycle (51, 52). Diatoms cope with low silica conditions by up-regulating silicic acid transporters such as *sit1* to maximize silica uptake (53–55). Indeed, strong induction of *P. australis*-expressed *sit1* was detected in metatranscriptomes in mid-June and early July (*SI Appendix*, Fig. S12), concurrent with the decrease in cell counts from the first phase of the bloom (Fig. 1*B*). Therefore, elevated *sit1* transcription may be informative for predicting bloom demise under silica-limiting conditions. All *sit1* genes expressed by *Pseudo-nitzschia* in this dataset are affiliated with clade A of diatom *sit* genes (56, 57) ( *SI Appendix*, Fig. S13, Table S4).

**Fig. 3.**
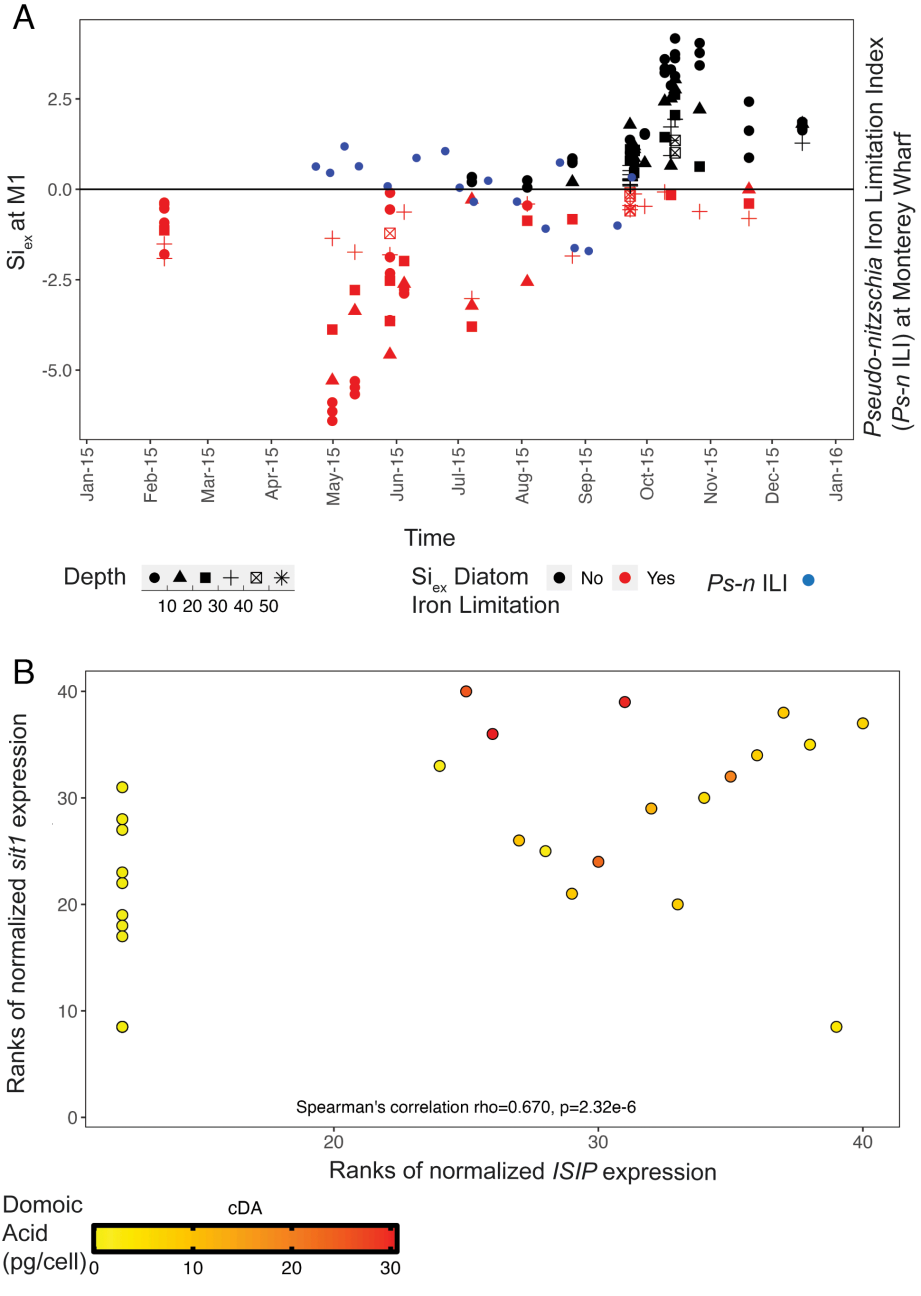
Iron and Si colimited the bloom and induced toxicity. (*A*) Negative values for Si_ex_, a proxy for diatom iron limitation, are present during the height of the bloom at Station M1 and co-occur with positive values of the *Pseudo-nitzschia* iron limitation index (*Ps-n* ILI) at Monterey Wharf, indicating iron limitation. As Si_ex_ trends positive later in the year, *Ps-n* ILI trends negative, indicating an alleviation of iron limitation. Si_ex_ was calculated in samples taken within the photic zone above 56 m depth. (*B*) Spearman ranks indicate that normalized *sit1* and *ISIP* are positively correlated (n = 48, Spearman’s rho: 0.670, *P*-value: 2.32e-6) and co-occur with higher per cell domoic acid. In order to highlight the response of *Pseudo-nitzschia australis* in relation to the total *Pseudo-nitzschia* community, raw read counts of *sit1* and *ISIP2A* and *ISIP2B* from *Pseudo-nitzschia australis* were normalized to total *Pseudo-nitzschia* read counts.

Silica limitation can be driven by iron limitation (58). Iron limitation causes diatoms to reduce iron-dependent nitrate uptake and meanwhile continue to take up silica, an iron-independent process, thereby drawing down dissolved silica concentrations. Previously unconsidered as a driver of the 2015 bloom, iron limitation was present in the Bay, as inferred by both macronutrient ratios and gene expression. Calculations of Si_ex_, a proxy for diatom iron limitation, suggest pervasive iron limitation throughout the photic zone of Monterey Bay early in the year and alleviation of iron limitation later in the year, as measured in outer Monterey Bay at Station “M1” and inner Monterey Bay at Station “C1” (Fig. 3*A* and *SI Appendix*, Fig. S14*E*) (59). Negative Si_ex_ values indicate that diatoms preferentially take up H_4_SiO_4_ relative to NO_3_^−^ due to iron deficiency, and more negative Si_ex_ values indicate a higher level of iron deficiency (59, 60). During the bloom, the mean flow of water in the Bay was from northwest to southeast, indicating prevalent advection from M1 toward MWII, and drifter data showed that Monterey Bay was strongly retentive of resident water and phytoplankton biomass (12). M1, in the path of Pt. Año Nuevo’s upwelling plume, is representative of changing conditions that affect the entire bay, including MWII (16). Gene transcription patterns corroborated iron limitation at MWII: Genes encoding iron starvation–induced protein (ISIP2A) were detected at MWII, indicating iron limitation. *ISIP* expression increased in early June during the relaxation of upwelling and persisted through the fall (*SI Appendix*, Fig. S15 and Table S5).

The resumed upwelling in late July to early August was also reflected in the nutrient composition at M1: Si_ex_ began to trend positive indicating an alleviation in iron limitation (Fig. 3*A*), likely due to a pulse of iron input in the upwelled water. Accordingly, *Pseudo-nitzschia* ferritin (FTN) expression, indicative of long-term iron storage, appeared in August (*SI Appendix*, Fig. S15 and Table S5). *Pseudo-nitzschia’s* FTN usage takes advantage of infrequent pulses of iron, which is beneficial during iron-limited conditions since they can utilize stored iron (61). Furthermore, the *Pseudo-nitzschia* iron limitation index (*Ps-n* ILI), trended from positive to negative in late July, indicating a switch from iron limited to iron replete *Pseudo-nitzschia* (62) (Fig. 3*A*). *Ps-n* ILI denotes *Pseudo-nitzschia* iron stress and limitation when the ratio of expression of ISIP2a to FTN is greater than the mean plus one SD of this ratio in iron-replete *Pseudo-nitzschia granii* laboratory cultures (62). Diatom nitrate transporters and ammonium transporters provide further evidence for regional upwelling conditions at MWII: Transporter expression peaked during upwelling conditions, both before and after the mid-summer decline in upwelling (*SI Appendix*, Fig. S16).

### Iron and Dissolved Silica Colimitation Actuated the Severe Bloom Toxicity.

Our metatranscriptomic data reveal that genes indicative of Si limitation (*sit1*) and iron limitation (*ISIP*) co-occur with each other and with higher per cell DA, suggesting that the *Pseudo-nitzschia* populations experienced nutrient colimitation, inducing DA production (Fig. 3*B*). Furthermore, in this dataset, *ISIP* expression must accompany *sit1* expression for cDA quotas to increase; in other words, if *sit1* is highly expressed, but there is no *ISIP* expressed, no DA is measured at the cellular level (Fig. 3*B*). These data imply that both Si limitation and iron limitation together were critical in inducing the high DA cell quotas observed during the 2015 HAB in Monterey Bay. As Fe’ bioavailability declines, Si:N also declines because silicification of diatoms increases and therefore, silica is limited secondarily (20). This phenomenon of increasing iron limitation and decreasing Si:N ratio has been previously described for diatom populations in the CCE and at a global scale (20, 58, 59). Previously overlooked, iron limitation may have played a significant role in driving the toxicity of the 2015 bloom in Monterey Bay and may have contributed to the Si limitation, both of which likely colimited the *Pseudo-nitzschia* population.

### Gene Transcription Predicts Domoic Acid One Week in Advance of Toxicity.

Predicting acute DA events is critical for management of fisheries and for protecting public health. This multiomic dataset was used as a model for molecular markers to predict DA toxicity. Gene expression is a relevant method for prediction given that transcription occurs temporally prior to protein translation and metabolite production. To implement a useful transcription-based model for HAB toxin modeling, it is important to set biologically relevant detection thresholds for host organism transcripts. Throughout most of 2015, reads mapping to *P. australis* transcripts could be detected (*SI Appendix*, Fig. S17; in 48 of 52 samples, *Pseudo-nitzschia*-mapping reads represented >0.1% of all reads mapped). Sharply decreased overall numbers of *P. australis*-mapped reads were detected during months when *Pseudo-nitzchia* was not detected in net tows (cell counts <10,000 per L). However, transcripts relating to housekeeping genes were still readily detected in samples with at least one thousand *Pseudo-nitzschia* per liter.

**Fig. 4.**
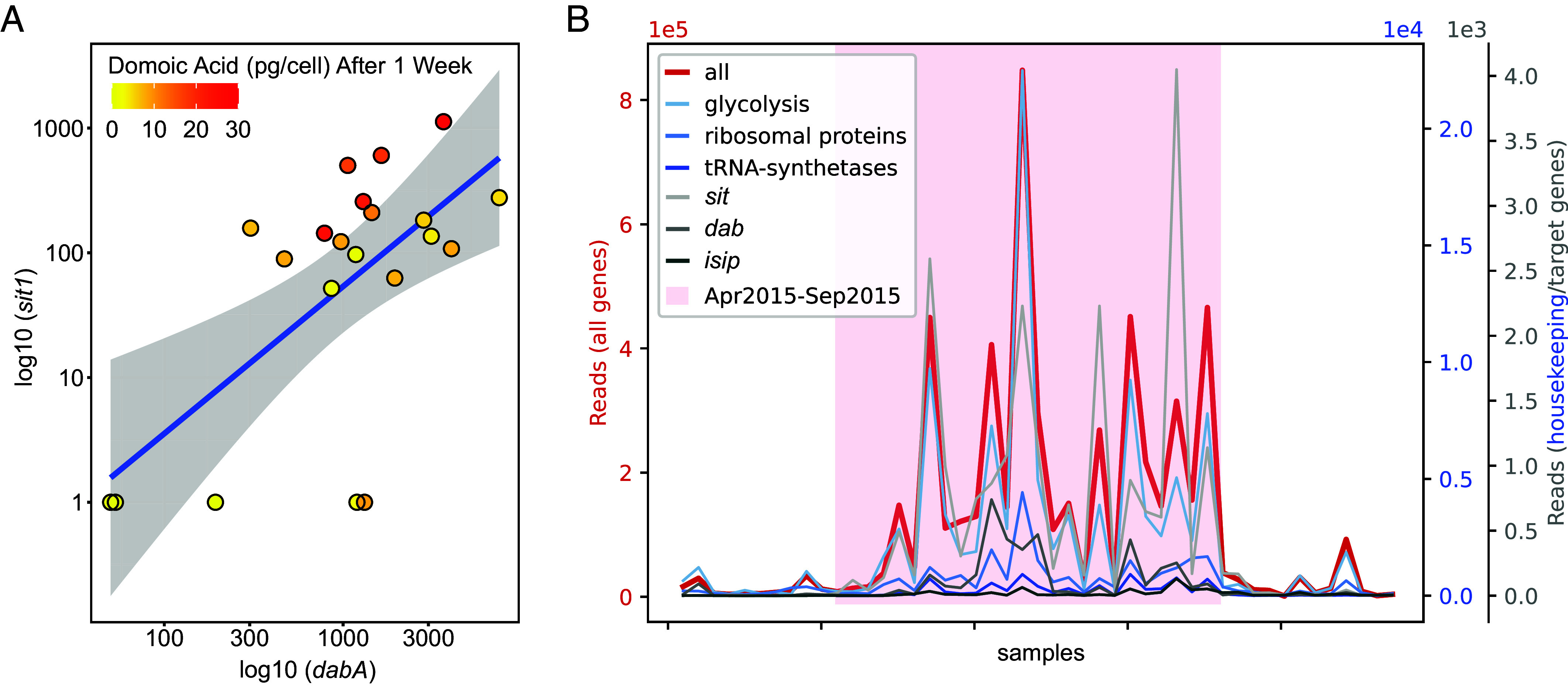
(*A*) Expression of *sit1* and *dabA* genes predict cellular domoic acid one week in advance of its production from April through September of 2015 (n = 27 data points). To highlight the response of *Pseudo-nitzschia australis* in relation to the total *Pseudo-nitzschia* community, raw read counts of *sit1* and *dabA* from *Pseudo-nitzschia australis* were normalized to total *Pseudo-nitzschia* read counts and multiplied by 1.0 × 10^6^. A constant of 1 was added to normalized *sit1* and *dabA*, and the axes were log_10_ transformed. Akaike information criterion (AIC) = 139.41, multiple R^2^ = 0.598, adjusted R^2^ = 0.543, *P*-value = 3.34e-4. Spearman’s correlation rho = 0.526, *P*-value = 1.44e-2. (*B*) *Pseudo-nitzschia* gene expression across year-round samples (n = 48 data points). Total (red) and specific read counts mapping to genes from several functional categories (shades of blue, gray) are plotted. Note that various categories have different y-axes (color-coded). The bloom season (April to September) with the highest gene expression is highlighted with a red background.

Given our previous analyses on toxin biosynthetic genes and nutrient biomarkers, we decided to focus on the interplay between *dabA* and *sit1*. Unlike *P. australis* housekeeping genes, expression of these two transcripts appears to be more constrained to active bloom months (*SI Appendix*, Fig. S17). We found that a generalized linear model of *P. australis* transcripts of *dabA* and *sit1* normalized to total *Pseudo-nitzschia* genus read counts predicts DA production one week in advance of its production during the active HAB event spanning April to September (Fig. 4*A* and *SI Appendix*, Fig. S18). Overall expression profiles of *dabA* and *sit1* confirm that elevated transcription of these two genes co-occurs with the bloom event and fluctuations in cell counts (Fig. 4*B*). Expression of these genes during bloom months predicts future DA levels a week in advance better than they predict contemporaneous DA levels (*SI Appendix*, Table S6). Further bloom events will need to be studied on similar or finer timescales to fully establish a sensitive forecasting model.

## Discussion

This study provides a robust molecular framework for understanding the progression, toxicity, and physiology of the 2015 *P. australis* HAB event in Monterey Bay, CA. Using 18S, 16S, and ITS2 amplicon sequencing, the transition from a *Chaetoceros*-dominated phytoplankton community to a nearly monospecific bloom of DA-producing *P. australis* was characterized. Due to the iron limiting conditions of the Bay early in the year as inferred by Si_ex_ and *Ps-n* ILI, molecular adaptations by *Pseudo-nitzschia* populations for long-term iron storage under low iron conditions likely enabled *Pseudo-nitzschia* to supersede and outcompete the upwelling-favored diatom *Chaetoceros* (61). Further study will be required to fully understand the competitive advantages that allowed for the dominance of *P. australis* over other toxic and nontoxic *Pseudo-nitzschia* species present before the monospecific bloom, such as *P. multiseries* and *P. fraudulenta*.

Iron limitation most likely contributed to both the competitive success of *Pseudo-nitzschia* and the elevated DA levels observed over several months in the region. Despite conflicting results regarding how iron limitation affects DA production (22, 63–67), we understand that actively growing blooms still in their exponential phase exhibit higher DA production under iron limitation (40). Iron limitation can mediate DA production and release. In other words, iron-stressed *Pseudo-nitzschia* cells are stimulated to produce 10 to 20 times more DA than non-iron-stressed *Pseudo-nitzschia*, and the presence of DA subsequently increases iron uptake by *Pseudo-nitzschia* as demonstrated in both lab and field studies (22, 65). In addition, iron-stressed *Pseudo-nitzschia* have been shown to release 95% of their DA extracellularly (65), a process that has been attributed to the potential role of DA as an iron chelator to bind iron and increase its bioavailability (64). One important caveat is that the relatively low iron-binding constant for DA necessitates an uncommonly elevated concentration of 100 nM of dissolved DA (dDA) for the molecule to compete with existing iron-binding ligands. Indeed, measurements of dDA around Monterey Bay exceeded 100 nM in our 2015 study (*SI Appendix*, Table S7), strongly suggesting that DA served as a relevant iron-binding ligand and provided *Pseudo-nitzschia* an advantage in acquiring bioavailable iron.

**Fig. 5.**
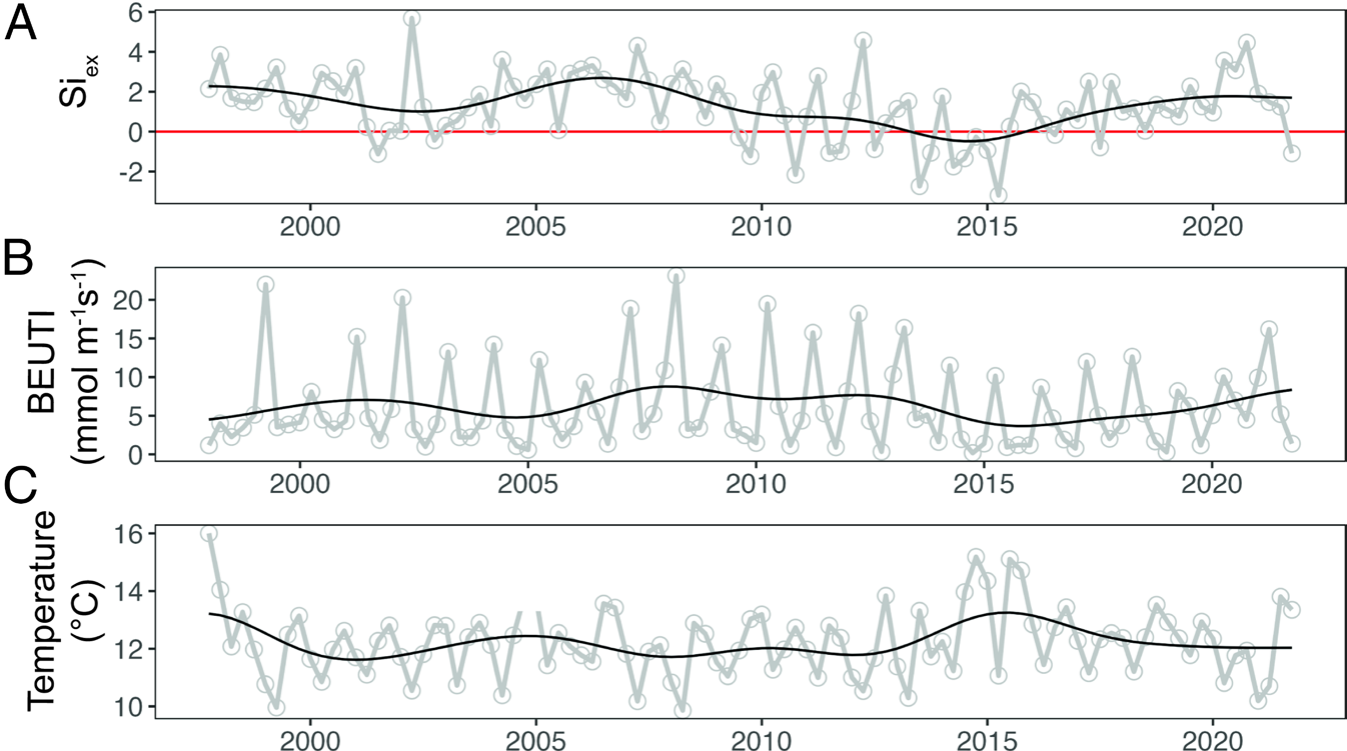
Historical seasonal averages from November 15, 1997, to November 15, 2021, of (*A*) Si_ex_ at M1 averaged from 0 to 40 m, (*B*) Biologically Effective Upwelling Transport Index (BEUTI) (https://oceanview.pfeg.noaa.gov/erddap/) from the North American West Coast at 37 °N latitude, (*C*) and temperature (°C) at M1 averaged from 0 to 40 m. To calculate Si_ex_, an R preformed value of 0.99 was utilized to estimate an average Si:N of upwelled water in the region. Si_ex_ values below the red line (y = 0) indicate time periods of diatom iron limitation.

DA events have been increasing in magnitude and intensity over the past several decades for reasons yet to be characterized (2, 68). We hypothesize that the increasing pervasiveness of iron limitation in the CCE is driving both the escalating prevalence of *Pseudo-nitzschia* and the exacerbation of toxin production (59). Global change is altering iron availability and distributions (69). Regions that experience more iron limitation will favor diatoms that use ferritin for long-term iron storage like *Pseudo-nitzschia*, and likely experience heightened DA production (61). Additional lower frequency variability in the CCE may play a role in increasing DA production potential. For instance, in 2015, both the Pacific Decadal Oscillation (PDO) and El Niño Southern Oscillation (ENSO) indices were in their positive phase, resulting in reduced alongshore winds, reduced upwelling, and lower available iron in the euphotic zone, causing the historically low Si_ex_ signature (Fig. 5 *A* and *B*). The high surface and subsurface heat content caused by the unprecedented marine heatwave beginning in 2014 further reduced the strength of upwelling (70, 71) (Fig. 5*C*). Monterey Bay has also been experiencing an increase in surface pCO_2_ and a decline in pH over the past twenty years, with eventual undersaturation of the system with respect to the carbonate ion and further acidification of upwelled waters due to equilibration with a higher pCO_2_ in the atmosphere as the expected outcome (72). Over the longer term, decreased pH will lower Fe’ uptake rates by ISIP2A, the predominant diatom iron-uptake mechanism, by reducing the availability of the carbonate binding cofactor (73). This trend toward increasing acidification is predicted to intensify iron limitation, and since DA production is correlated with iron limitation and acidification, future world oceans may bear witness to heightened DA (65, 74).

Active *dab* transcription is inextricably linked to accumulation of DA, and we found that transcription of *dabA* and *sit1* predict DA a week in advance of its production during this bloom. For optimization of field applications, *P. australis dabA* normalized to the cell counts of *Pseudo-nitzschia “*seriata” size class may become a highly predictive and easy-to-use measurement for anticipating DA events with one week lead-time. Additional testing of the predictive power of this model on blooms of various toxicity levels in several geographic regions along with false negatives and false positives will be needed prior to application. However, if implemented, such an approach could be effectively deployed by coastal monitoring groups and resource managers as an early warning system that only requires a qPCR assay for *dabA* in addition to the already-collected *Pseudo-nitzschia* cell counts. Nothing like this currently exists, and in fact, many monitoring programs are generally hindered by long lags in chemical analysis of DA that limit the benefits of real-time observing. Metatranscriptomic sequencing of *Pseudo-nitzschia* bloom events, including more frequent sampling on the timescale of days rather than weeks, will be necessary to fine-tune the timeline for *dab* detection to better inform bloom forecasting. Additionally, the precise nucleotide and translated amino acid sequences of *dab* transcripts lend insight into DA producing species. Given the paucity of *dab* transcripts currently identified, further sequencing of *dab* genes and transcripts will be required to fully explore this trend among all DA-producing *Pseudo-nitzschia* species and isolates. Additionally, induced expression of silica-responsive genes such as *sit1* may reflect active diatom silica limitation or starvation and may help predict impending bloom demise.

The metatranscriptomic approach employed in this study has also enabled preliminary insight into the changing physiology of *Pseudo-nitzschia* HABs from inception to demise. We described trends in the relative expression of genes involved in active growth, photosynthesis, toxin production, and environmental stress. A targeted, Lagrangian sampling of surface waters or subsurface chlorophyll maxima throughout a bloom event may yield improved functional clustering of gene expression by following discrete *Pseudo-nitzschia* subpopulations in the water column (75). Models for *Pseudo-nitzschia* HAB physiology will be improved further if Lagrangian sampling is conducted with higher frequency.

This study represents a comprehensive molecular description of a major eukaryotic marine HAB event. We have created a robust framework for studying HAB events by connecting routine monitoring data, such as toxin measurements and phytoplankton cell counts to molecular HAB species identification and toxin biosynthesis gene expression. The synthesis of these data provides unique insights into *Pseudo-nitzschia* HAB physiology as it relates to nutritional and physical conditions and provides an improved molecular framework for HAB monitoring and prediction. The continued study of molecular physiology in HABs promises to lend essential insight into the nature of toxin production and bloom formation in the changing world ocean.

## Materials and Methods

Method details are available in *SI Appendix*. All routine monitoring data collected weekly from Monterey Municipal Wharf II (MWII) during the study period, including *Pseudo-nitzschia* cell counts, DA measurements, local chlorophyll concentration, temperature, and nutrient data, is made publicly available through the Southern California Coastal Ocean Observing System (SCCOOS, https://sccoos.org/harmful-algal-bloom/) as part of the California Harmful Algal Bloom Monitoring and Alert Program (CalHABMAP).

RNA was extracted using a modified version of the Direct-zol RNA Miniprep Plus kit (Zymo Research), and cDNA was synthesized using the SuperScript III First-Strand Synthesis System (Life Technologies). Poly-A enriched RNA was synthesized using the TruSeq Stranded mRNA prep kit (Illumina). Amplicons were sequenced on the MiSeq PE300 (Illumina), and the poly-A enriched RNA on the HiSeq4000 PE150 platform (Illumina). Sequence data have been deposited in the NCBI Sequence Read Archive under accession number PRJNA1027375. Sample metadata, assembly, and open reading frames are available on Zenodo (https://zenodo.org/records/10728894).

## Supplementary Material

Appendix 01 (PDF)

Dataset S01 (CSV)

## Data Availability

Sequence Data have been deposited in NCBI (PRJNA1027375) (76). All study data are included in the article and/or Supporting Information.
