# Supplementary material for: Molecular forecasting of domoic acid during a pervasive toxic diatom bloom
Source: Proc Natl Acad Sci U S A. 2024 Sep 19;121(40):e2319177121. doi: 10.1073/pnas.2319177121 (PMC11459128; doi:10.1073/pnas.2319177121)
Supplement: Supplementary file 1 — Appendix 01 (PDF) [file pnas.2319177121.sapp.pdf]

Supporting Information for

Molecular Forecasting of Domoic Acid during a Pervasive Toxic Diatom Bloom

John K. Brunson[a,b,1], Monica Thukral[a,b,1], John P. Ryan[c], Clarissa R. Anderson[a], Bethany C. Kolody[a,d], Chase James[e], Francisco P. Chavez[c], Chui Pin Leaw[f], Ariel J. Rabines[a,b], Pratap Venepally[b], Zoltan Fussy[a,b], Hong Zheng[b], Raphael M. Kudela[g], G. Jason Smith[h], Bradley S. Moore[a,i], Andrew E. Allen[a,b,2]

[1]These authors contributed equally to this work.

[2]To whom correspondence may be addressed. **Email**: aallen@ucsd.edu

This PDF file includes:

- Supporting text, including Materials and Methods
- Figures S1 to S20
- Tables S1 to S8
- SI References

Other supporting materials for this manuscript include the following:

- Dataset S1: *Pseudo-nitzschia* ORFs Dataset

Supporting Information Text

Materials and Methods

Routine Monitoring Data Availability and Visualization of Satellite Data

All routine monitoring data collected weekly from Monterey Municipal Wharf II (MWII) during the study period, including *Pseudo-nitzschia* cell counts, DA measurements, local chlorophyll concentration, temperature, and nutrient data, are made publicly available through the Southern California Coastal Ocean Observing System website (SCCOOS, https://sccoos.org/harmful-algal-bloom/) as part of the California Harmful Algal Bloom Monitoring and Alert Program (CalHABMAP). Satellite data were acquired and visualized via the Environmental Research Division's Data Access Program (ERDDAP) data server from the National Oceanic and Atmospheric Association (NOAA). All-surface temperature data were collected in Local Area Coverage (LAC) format by the Advanced Very High Resolution Radiometer (AVHRR) scanner onboard NOAA's Polar Orbiting Environmental Satellite (POES). Satellite-measured remote chlorophyll concentration (mg/m^3) was collected by the Visible Infrared Imaging Radiometer Suite (VIIRS) onboard the Suomi National Polar-Orbiting Partnership (Suomi NPP) satellite.

Phytoplankton Net Tow and Sample Filtering

Weekly net tows from MWII were obtained using a 20 cm diameter, 20 µm mesh net to concentrate surface waters to a depth of five meters, as is routine for monitoring at MWII. From the net tows, 100 mL of concentrated phytoplankton samples were applied to 0.22 µm polycarbonate (PC) filters, which were then stored in 1 mL of TRIzol (Invitrogen) reagent in 15 mL conical tubes, mixed by vortexing, and kept at -80°C for future processing. A total of 52 samples were processed and stored in this manner.

RNA extraction and cDNA generation

Samples were removed from -80°C storage. The PC filter was removed from the TRIzol solution and samples were subsequently centrifuged for 20 minutes at 2500 x g and 4 ^{o}C to pellet particulate matter. The supernatant was then transferred to a new tube. RNA extraction begins with the addition of 200 µL of chloroform to each sample, which were then shaken vigorously for 15 seconds and allowed to stand for 12–15 minutes at room temperature (~25 ^{o}C). The resulting mixture was then centrifuged at 12,000 xg for 15 minutes at 4 ^{o}C. Next, the aqueous phase was transferred to a new tube, adding an equal volume of 100% ethanol and mixing well. The mixture was then transferred to a Zymo-Spin IIICG Column (Zymo Research), after which the RNA extraction was completed using the Direct-zol RNA Miniprep Plus kit (Zymo Research) following the manufacturer protocol, including an on-column DNA digestion step using DNase I (New England Biolabs). Suitable RNA quality was verified using an Agilent 2100 Bioanalyzer.

Synthesis of cDNA to be used as template for generation of 18S-V4, 16S and ITS2 amplicons was preformed using the SuperScript III First-Strand Synthesis System (Life Technologies). Following kit standard protocol, 100 ng of total RNA per sample was used with random hexamer primers to make 20 µL of cDNA.

Preparation and sequencing of 18SV4, 16S, and ITS2 amplicon libraries

Amplicon libraries for 18SV4, 16S and ITS2 sequences were generated using the cDNA libraries from above as a template for the one-step PCR reactions to simultaneously amplify target sequences and incorporate Illumina adaptors, linker sequences, and unique barcoded indices. All one-step PCR reactions were performed using the TruFi DNA Polymerase PCR kit (Azura). All primers used in this study, together with attached adaptors, linkers and barcoded indices are listed in Table S8. The 18SV4 region was amplified using the primer pair V4F and V4RB (1). The 16S(V4-V5) region was amplified using the primer pair 515F-Y and 926R (2). The ITS2 region was amplified using primer pair 5.8SF and 28SR (Table S8). Primer pairs containing unique combinations of barcoded indices, allowing downstream demultiplexing, are generated in 10 µM concentration and stored in 96-well plates.

PCR reactions are then set up using 1 µL of cDNA as template, 0.4 µM of primer pair, and TruFi DNA Polymerase and buffer mix per manufacturer protocol and brought to a total reaction volume of 25 µL using molecular grade water. PCR reactions for 18SV4 and 16S amplicon generation were performed with an initial denaturing step at 95°C for one minute followed by 30 cycles of denaturing at 95°C for 15 seconds, annealing at 56°C for 15 seconds, and extension at 72°C for 30 seconds. Initial PCR reactions for ITS2 followed a similar protocol, however improved amplification was observed when the annealing temperature was decreased to 51-53°C. To confirm amplification and correct size of amplicon, 2.5 µL of each PCR reaction was run on a 1.8% agarose gel.

The PCR products were then cleaned up using AMPure XP beads (Agencourt) and the standard PCR purification protocol per manufacturer recommendations and eluted in 35 µl of elution buffer. Cleaned PCR products were then quantified using the PicoGreen Quant-IT dsDNA Quantitation Reagent (Life Technologies). Equal quantities of 18SV4, 16S or ITS2 amplicons (~10 ng per reaction) were pooled, cleaned, and concentrated using AMPure XP beads using standard protocol, eluting in 45 µl of elution buffer. Final library quality was assessed using a TapeStation (Agilent) and quantified on a Qubit fluorometer (ThermoFisher). Respective pools were then sequenced on the MiSeq PE300 (Illumina) generating 250 bp paired end reads for all amplicon libraries. Sequencing was performed at the UC Davis Sequencing Core.

Assembly, taxonomic classification and visualization of amplicon sequencing

The 18SV4, 16S and ITS2 amplicon sequences were imported separately into Qiime2 version 2019.4.0 where the dada2 plug-in was used to merge, quality filter the reads, identify and remove chimeric reads, and generate Amplicon Sequence Variants (ASVs) and count tables (3, 4). Taxonomy was assigned to the 18S ASVs using a qiime2 naïve-bayes classifier trained on the PR2 version 4.11.1 database (5). The 16S ASVs were annotated first using a qiime2 naïve-bayes classifier trained on the Silva-132 database in order to differentiate bacterial and mitochondrial sequences from chloroplast sequences (6). Chloroplast ASVs were extracted in Qiime2 and further annotated using a qiime2 naïve-bayes classifier trained on the PhytoREF database (7). Genus and species classifications for ITS2 ASVs were assigned using the "classify-consensus-blast" feature classifier, using a database of *Pseudo-nitzschia* ITS2 sequences as the reference BLAST database (8). Conglomeration of ASV counts by taxonomic level and subsequent generation of stacked bar plots was performed in PhyloSeq v.1.38.0 (9).

Preparation and sequencing of polyA-enriched RNA

Starting with 500 ng of total RNA per sample, we used the TruSeq Stranded mRNA prep kit (Illumina) to make polyA-enriched libraries suitable for RNA sequencing, following manufacturer protocols. Following preparation of polyA-enriched libraries, samples were combined into two pools and ran on the HiSeq4000 PE150 platform (Illumina) at the UC Davis Sequencing Core.

Assembly and annotation of RNA sequencing libraries

The resulting demultiplexed HiSeq4000 read libraries were processed via the RNAseq Annotation Pipeline v0.4 (10). Reads were first trimmed for quality and filtered to remove primers and adaptors using an in-house trimmer (Table S1, qtrim_min_len=30, qtrim_min_quality=33), and rRNA sequences using riboPicker v.0.4.3 (-dbs rrnadb -z 3) (11). CLC Genomics Workbench 9.5.3 (QIAGEN) was used to assemble contigs by library, then a global assembly was compiled by clustering the library assemblies using CD-HIT v4.6.1 (-c 0.95 -mask NXWSMKRYBDHV). Open reading frames (ORFs) were predicted from the assembled contigs using FragGeneScan v1.31 (-complete=0) (12). The resulting ORFs were automatically annotated for function via BLASTP v2.2.26 searches (-e 1e-03) against the KEGG and KOG databases, and via HMMER v3.0 searches (--cut_tc -Z 15930) against thePfam and TIGRFAM databases. ORF taxonomy was inferred based on the best BLAST hits against the reference dataset PhyloDB 1.076 consisting of protein sequences from 1,108 eukaryote, 5,713 bacterial, 282 archaeal and 20,097 viral species (-e 1e-03) (10, 13). Trimmed reads were mapped back to assembled contigs using clc_ref_assemble_long of the CLC Genomics Workbench to generate read counts (14).

Phylogeny of *sit1* genes

Protein sequences were aligned with the reference set from Durkin *et al.* (2016) using MUSCLE v5.1 (-super5; Edgar 2022), the alignment trimmed using trimal (-gt 0.3; Capella-Gutiérrez *et al.* 2009), and their phylogeny inferred using IQ-TREE v2.2.6 with LG+G4 substitution matrix, and with support values calculated using SH-aLRT and ultra-fast bootstrapping (1000 replicates each; Minh *et al.* 2020) (15–18).

Defining expression modules for the *P. australis* bloom

The Weighted Gene Correlation Network Analysis (WGCNA) R package, version 1.72.1, was implemented to identify modules of *P. australis* ORFs with similar expression patterns to define functional clusters (19). The bloom period for WGCNA analysis purposes was defined as 15th April through 30th September of 2015. *P. australis* ORFs were identified and filtered based on taxonomic annotation above, leveraging the PhyloDB 1.02 database which contains a *P. australis* annotated transcriptome (10, 20). This yielded 14873 *de novo* assembled *P. australis* ORFs. To obtain statistically relevant results, only ORFs with 10 or more reads in 80% of the libraries comprising the bloom period were considered, yielding 437 ORFs for WGCNA analysis.

Following the filtering step, expression was normalized by dividing reads mapped to a given ORF by the total number of reads mapped to all assembled *P. australis* ORFs to account for library and *P. australis* population size prior to WGCNA analysis and construction of a correlation matrix. An adjacency matrix was built from the correlation matrix input by applying a power function (AF(s)=s^b) to the input data, where "b" is defined as the soft-thresholding parameter. A soft-thresholding parameter of b = 6 was found to be lowest value at which a scale-free topology R^2 value exceeding 0.8 was achieved (Fig. S19). This information was used to construct the consensus matrix and dendrogram using the function "blockwiseConsensusModules" using the parameters: b = 6, TOMType = "signed", detectCutHeight = 0.995, reassignThreshold = 0 (Fig. S20). The "moduleEigengenes" function was used with default parameters to select an "eigengene," or the ORF whose expression profile is most representative of the other profiles contained in the module. No minimum module size was set and no modules were merged to generate modules containing genes with the highest possible similar expression profiles to one another, maximizing the variance explained by each eigengene.

Analysis of markers for iron limitation and DA prediction

Si_{ex} was calculated as the concentration of silicate minus the concentration of nitrate times the ratio of H_4SiO_4 to NO_3^- at the regional upwelling source depth (21). Si_{ex} was calculated assuming that the ratio (µmol/L:µmol/L) of H_4SiO_4 to NO_3^- at the regional upwelling source depth is equal to 1.

Pseudo-nitzschia iron limitation index (*Ps-n* ILI) was calculated from equations based on the expression of ISIP2a relative to FTN of laboratory-grown *P. granii* cells (22). *Ps-n* ILI calculations were preformed using normalized gene expression in transcripts per million (TPM) rather than raw read counts. Samples were not omitted if ferritin expression was zero.

To evaluate whether sample sequencing depth allowed efficient target transcript detection, we determined the average transcription rates of three groups of housekeeping genes (glycolytic enzymes, ribosomal and translation initiation proteins, tRNA-synthetases) and compared their expression with *sit*, *dab*, and *isip2*/ferritin genes in four periods of sampling (January–March, April–June. July–September, October–December). A pseudocount (10^{-3}) was added to all expression values prior to average calculation. We observed that some genes were undetected off-bloom (January–March/October–December), but almost all genes were detectable in the bloom season (April–September) (Figure S17). Overall, the data suggested sufficient sequencing coverage in bloom to analyze iron/silicate starvation marker genes. Importantly, target genes often showed comparable transcription rates as housekeeping genes.

Linear regressions were conducted using glm() and lm() functions in the stats package (version 3.6.2) in RStudio (version 2022.02.3). Generalized linear models were cross-validated against original data with the cv.glm() function of the boot package (version 1.3-28.1). Gene expression raw read counts from *Pseudo-nitzschia australis dabA* and *sit1* contigs were normalized to total *Pseudo-nitzschia* read counts for analysis. Contig IDs for *P. australis dabA* and *sit1* can be found in Tables S3 and S4.

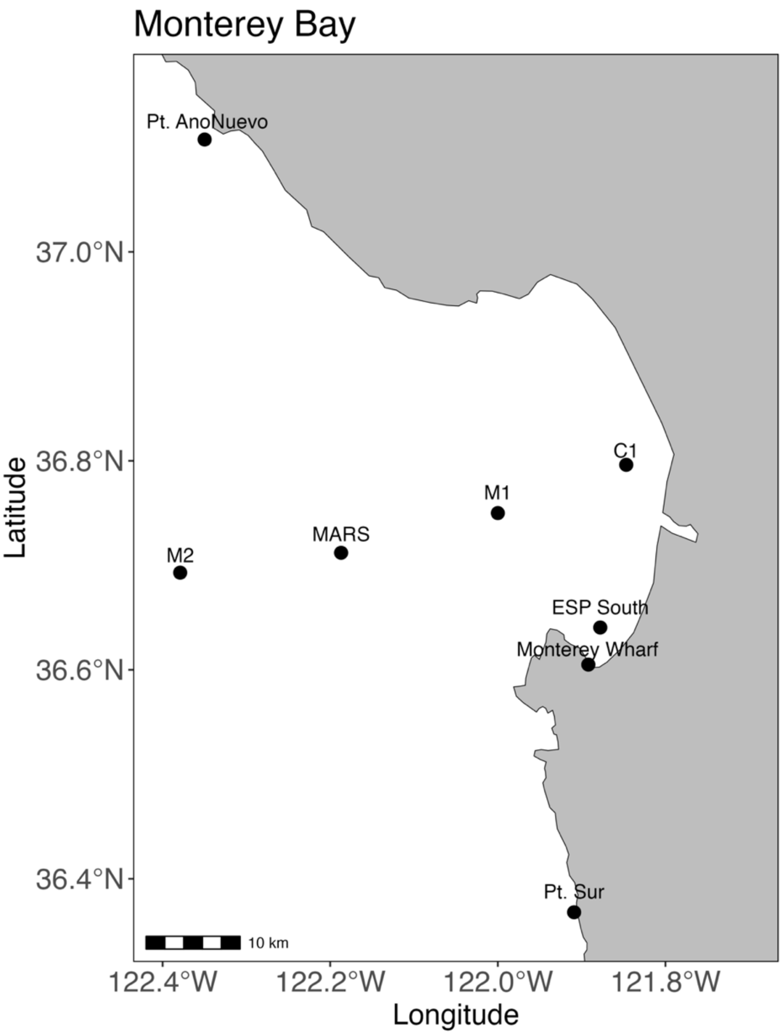

Figure S1. Sampling location at Monterey Wharf II (MWII) is marked together with the upwelling centers outside the bay, Pt. Año Nuevo and Pt. Sur. Also marked are regional monitoring locations used for Si_{ex} analysis.

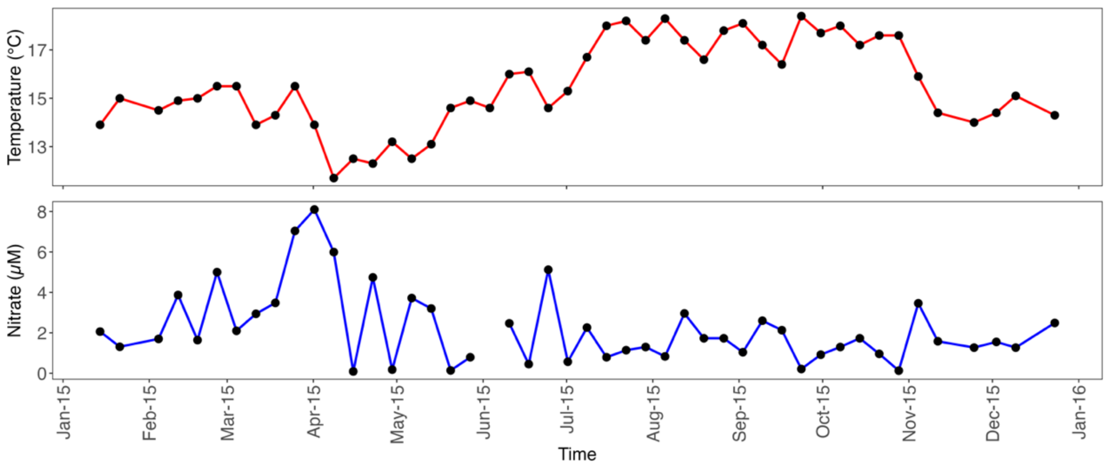

Figure S2. (Top) Temperature (°C) and (Bottom) nitrate (mM) measurements collected from MWII.

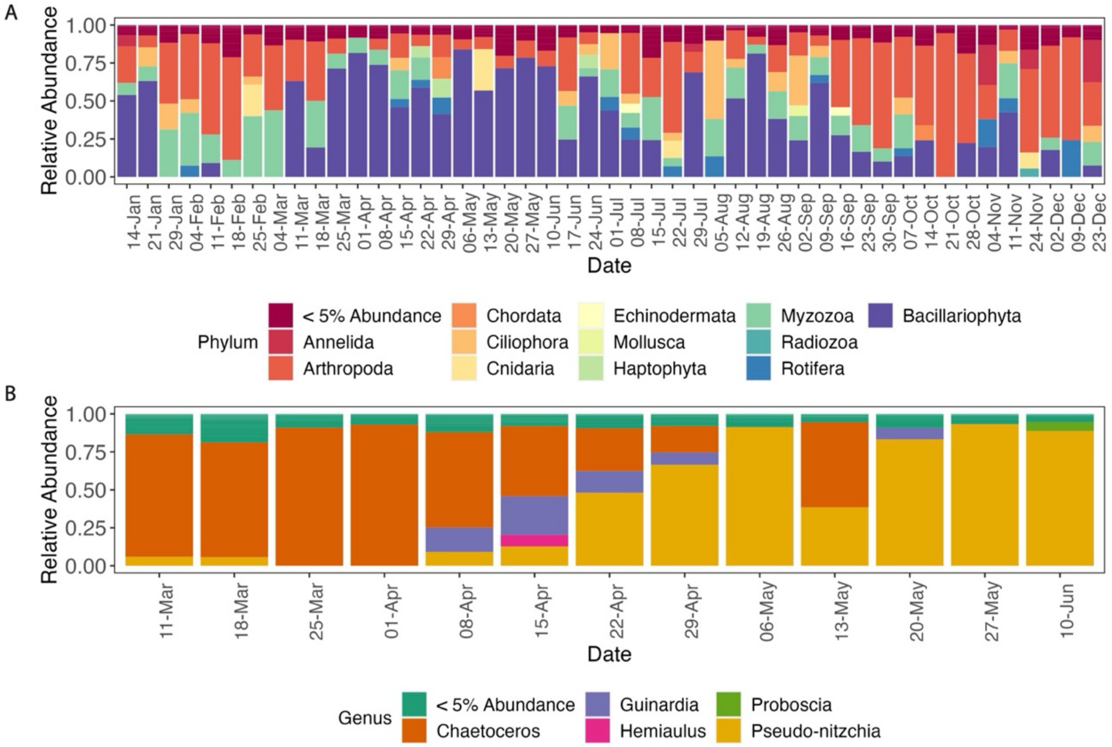

Figure S3. 18S-V4 Amplicon Sequencing of 2015 MWII Samples. (A) Relative abundance of phyla in 18S-V4 amplicon sequencing libraries, (B) Relative abundance of diatom (Bacillariophyta) genera during the spring phytoplankton succession.

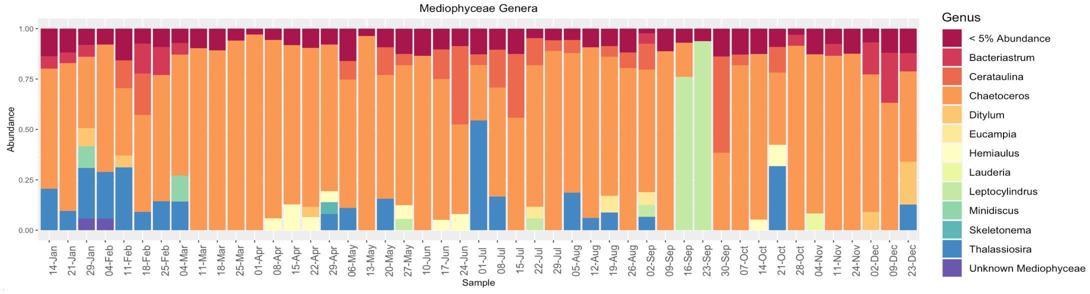

Figure S4. Genus composition of Mediophyceae (centric) diatoms determined by 18SV4 sequencing from weekly samples throughout 2015.

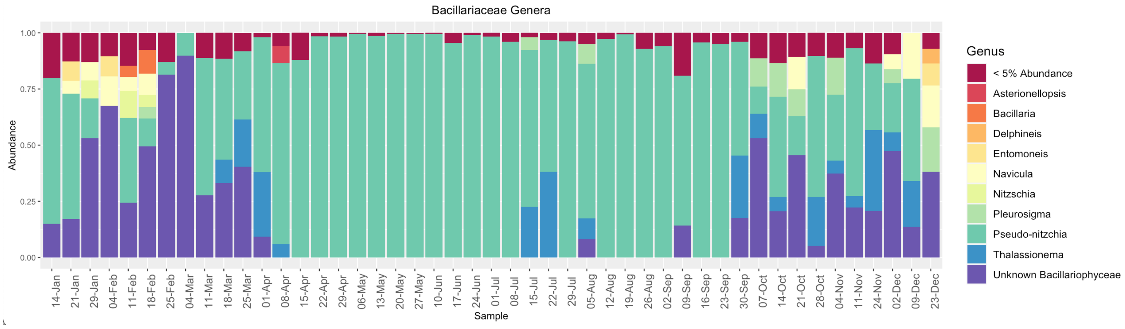

Figure S5. Genus composition of Bacillariophyceae (pennate) diatoms determined by 18SV4 sequencing from weekly samples throughout 2015.

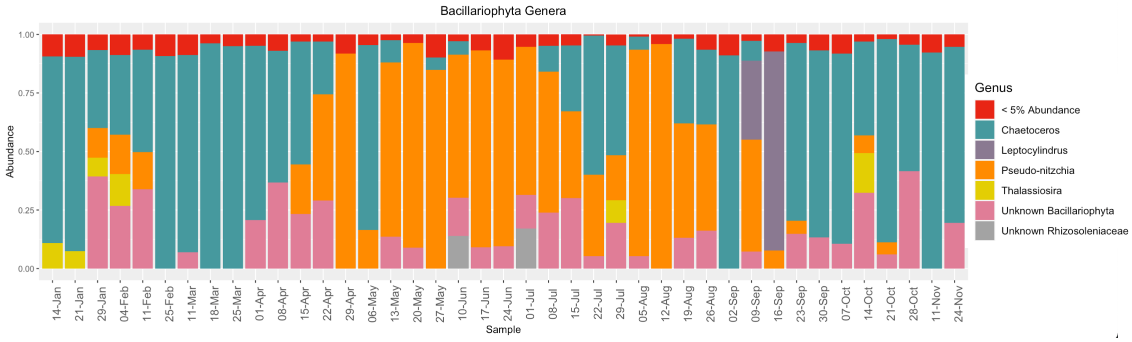

Figure S6. Genus composition of diatoms (Phylum Bacillariophyta) determined by 16S chloroplast sequencing from weekly samples throughout 2015.

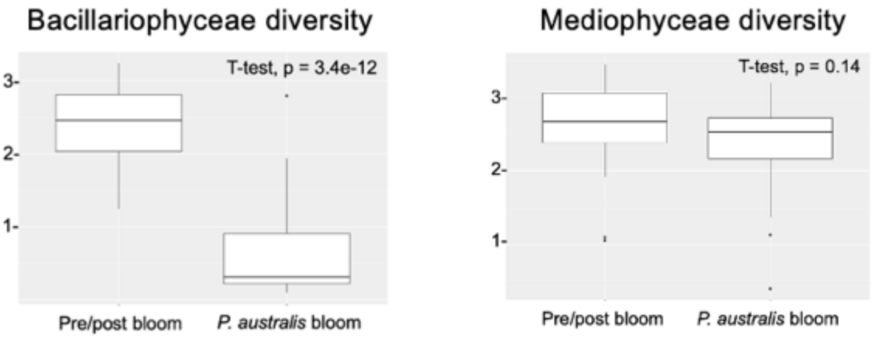

A **B**

Figure S7. 18S-V4 Amplicon Sequencing of 2015 MWII Samples. (A) Shannon alpha-diversity of pennate (Bacillariophyceae) diatoms comparing *P. australis* bloom samples (Apr 22nd – Sep 30th) with non-bloom samples (rest of year). (B) Shannon alpha-diversity of centric (Mediophyceae) comparing bloom samples (Apr 22nd – Sep 30th) with non-bloom samples (rest of year).

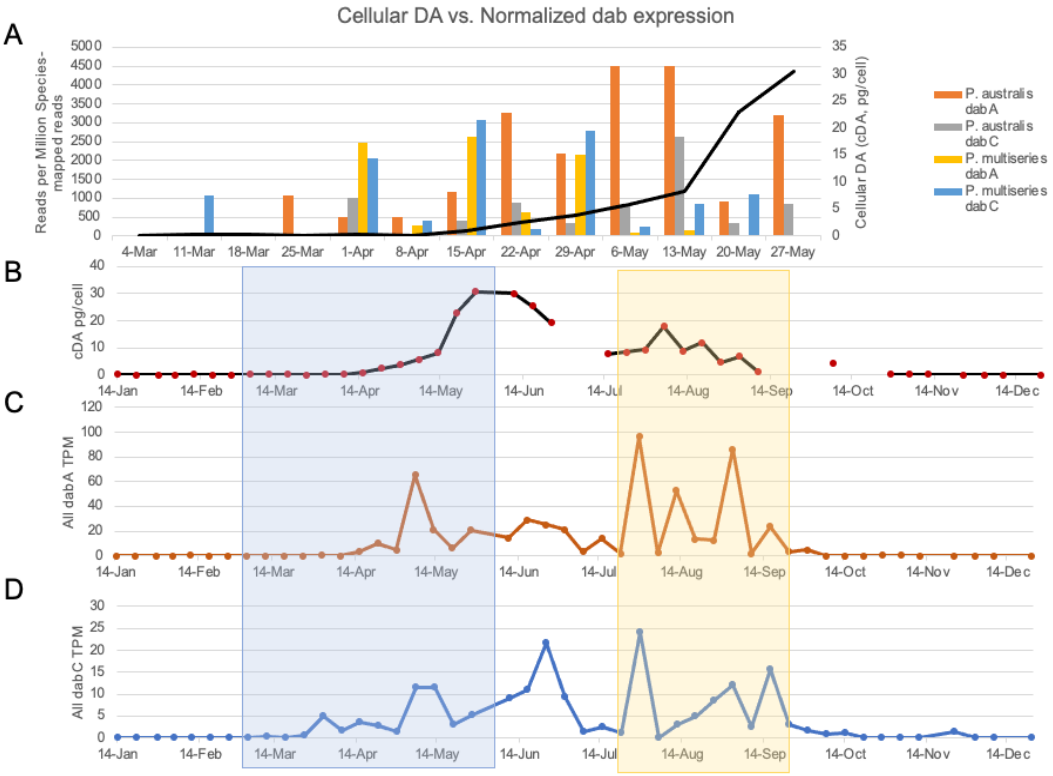

Figure S8. Transcription of *dabA* and *dabC* throughout the bloom. (A) Normalized expression of *dabA* and *dabC* transcripts plotted against cellular DA (cDA, pg/cell) during bloom initiation in the spring. Assigned reads for *P. australis* or *P. multiseries dab* transcripts were normalized by the total number of *P. australis* or *P. multiseries*-mapped reads per library to estimate relative expression within each DA-producing species, (B) cDA values for the 2015 calendar year, (C) Summed expression of all *dabA* transcripts, represented as library normalized per-million reads, (D) Summed expression of all *dabC* transcripts, represented as library normalized per-million reads. Early and late bloom phases indicated in (B-D) with blue and orange boxes, respectively.

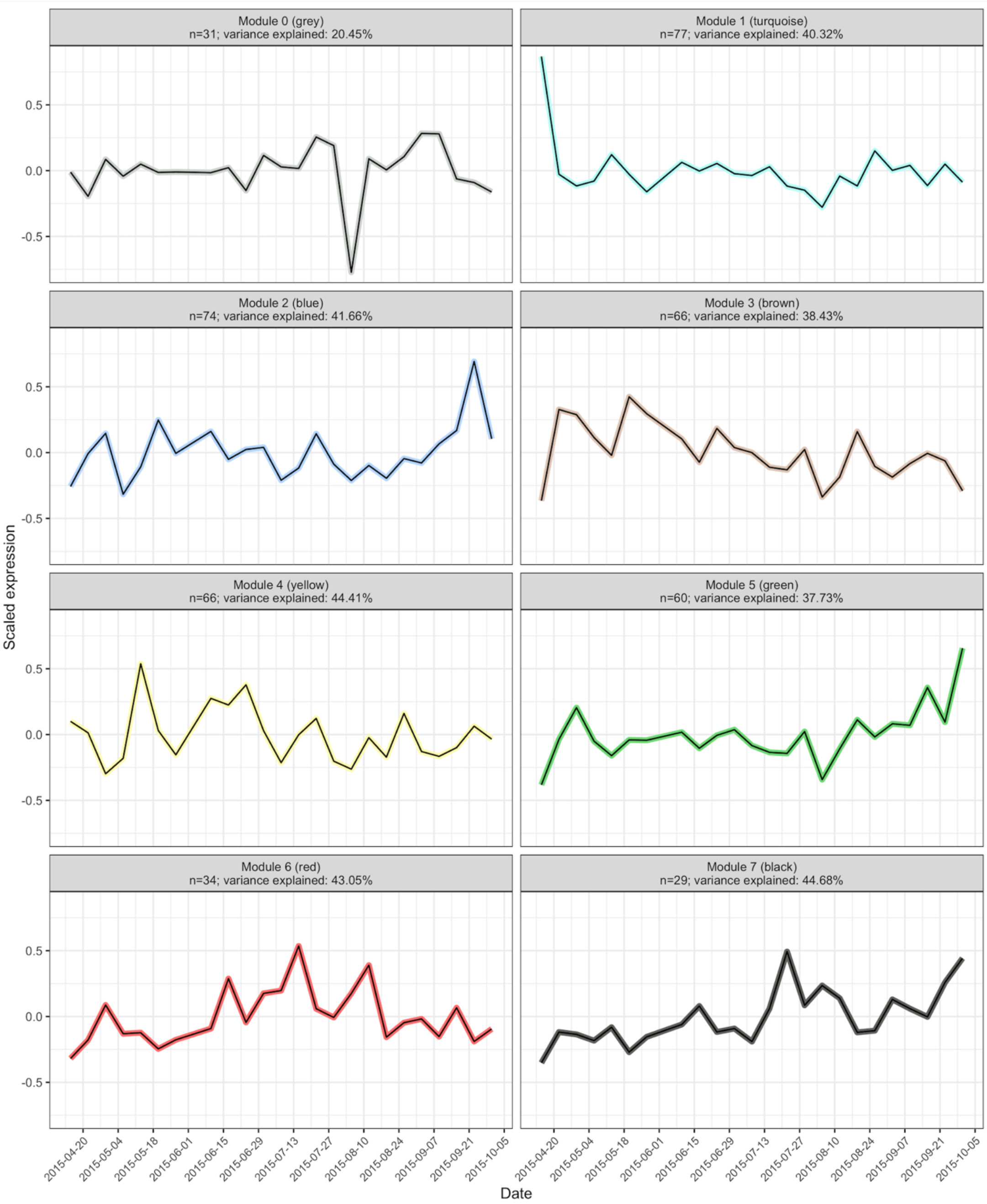

Figure S9. Relative expression modules for *P. australis* transcripts throughout the HAB event determined by WGCNA. Weighted gene correlation network analysis (WGCNA) on a highly-expressed subset of the *de-novo* assembled *P. australis* HAB metatranscriptome identified eight modules of transcripts with similar expression profiles (modules 1-7), including one module of remaining contigs with low overall variance explained by the model (module 0).

A

DA or isoprenoid biosynthesis gene names	WGCNA Module
**dabA**	Module 7
**dabC**	Module 4
**dabD**	Module 7
**HDS**	Module 7
**IDI**	Module 7
**DXS**	Module 2
**HDR**	Module 4

B

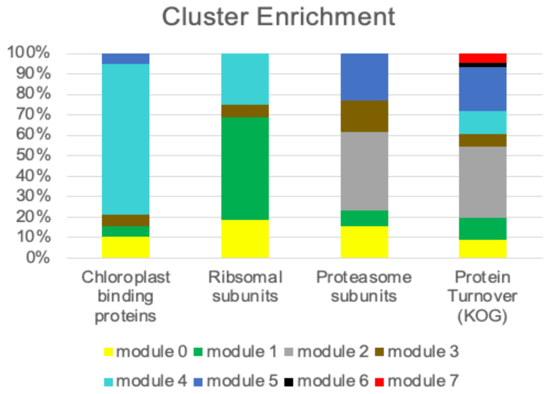

Figure S10. Annotations of interest from WGCNA clustering analysis (*A*) Functional clustering of DA biosynthesis (*dab*) and isoprenoid biosynthesis transcripts. (*B*) Enrichment of ORF annotations in WGCNA modules. Chloroplast binding proteins, ribosomal subunits and proteasome subunits were determined on the basis of PFam annotation, among other annotations. “Protein turnover (KOG)” ORFs includes all proteins in the KOG class “Posttranslational modification, Protein turnover, Chaperones.”

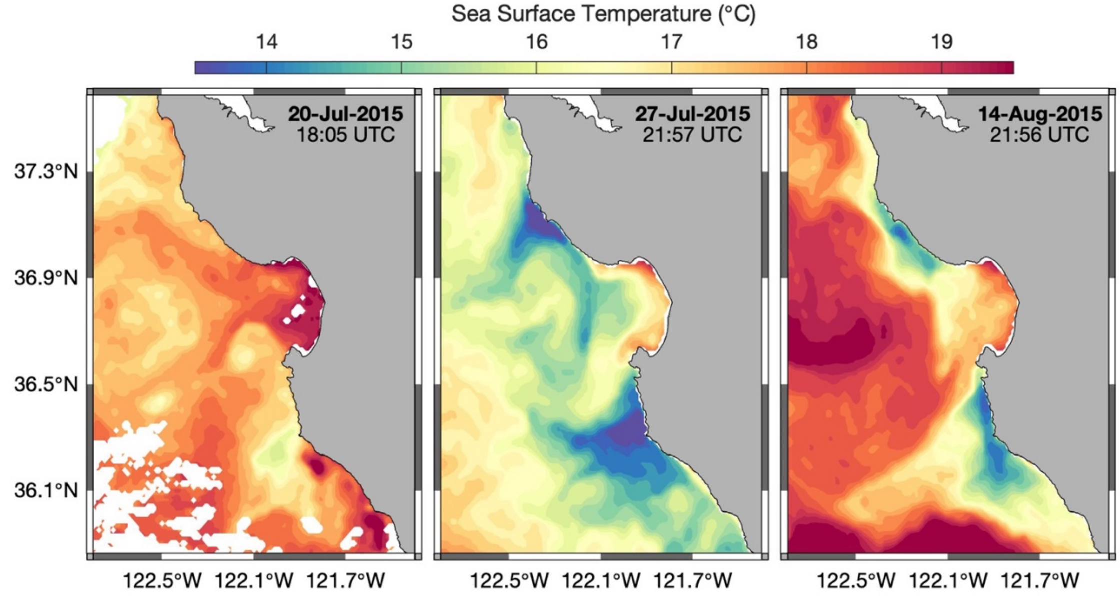

Figure S11. Satellite imagery of Monterey Bay illustrating upwelling (cool plumes) during late-July and mid-August. Sea surface temperature (°C) of Monterey Bay on (left) 20-July (middle) 27-July, and (right)14-August from NOAA AVHRR.

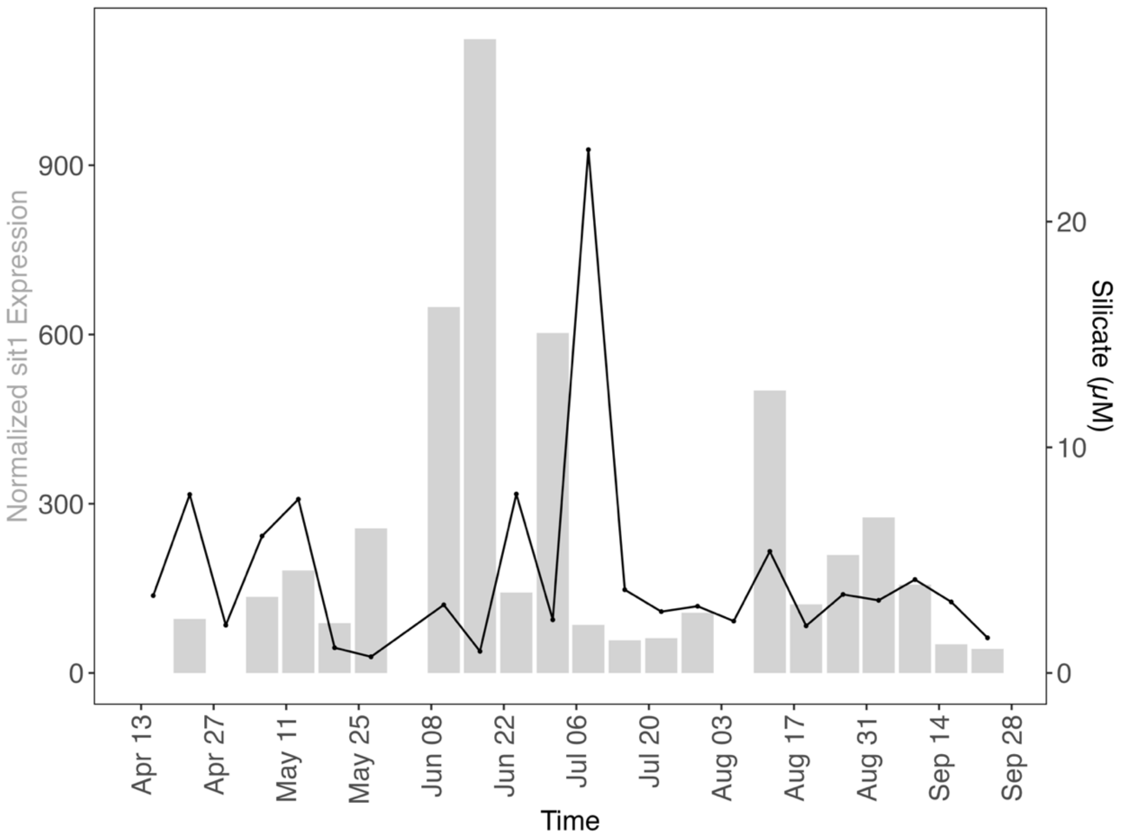

Figure S12. Normalized expression of *P. australis sit1* (gray bars) compared to dissolved silica concentrations (black) at MWII during the HAB. Raw read counts of *sit1* from *Pseudo-nitzschia australis* were normalized to total *Pseudo-nitzschia* read counts and multiplied by 1.0 x 10^6.

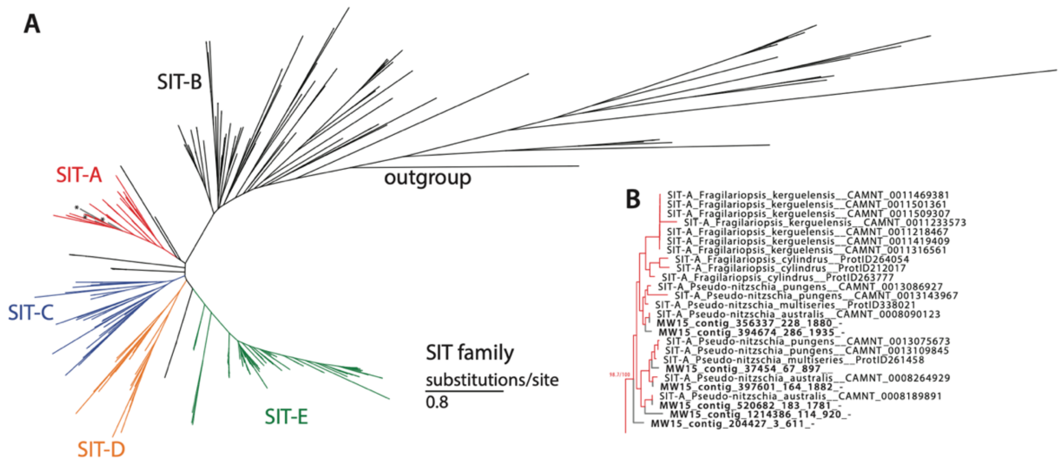

Figure S13. Phylogenetic relationship of Monterey Wharf 2015 *Pseudo-nitzschia* silicon transporters. (A) Maximum likelihood phylogeny with a reference set of proteins from Durkin *et al.* (2016; 675 amino acid positions) depicts the affiliation of the *Pseudo-nitzschia* sequences with clade A (15). The positions of the seven ORFs from this study are marked with asterisks. (B) A subset of the tree showing the position of the seven ORFs. Only the support for the *Pseudo-nitzschia-Fragilariopsis* clade is shown for clarity.

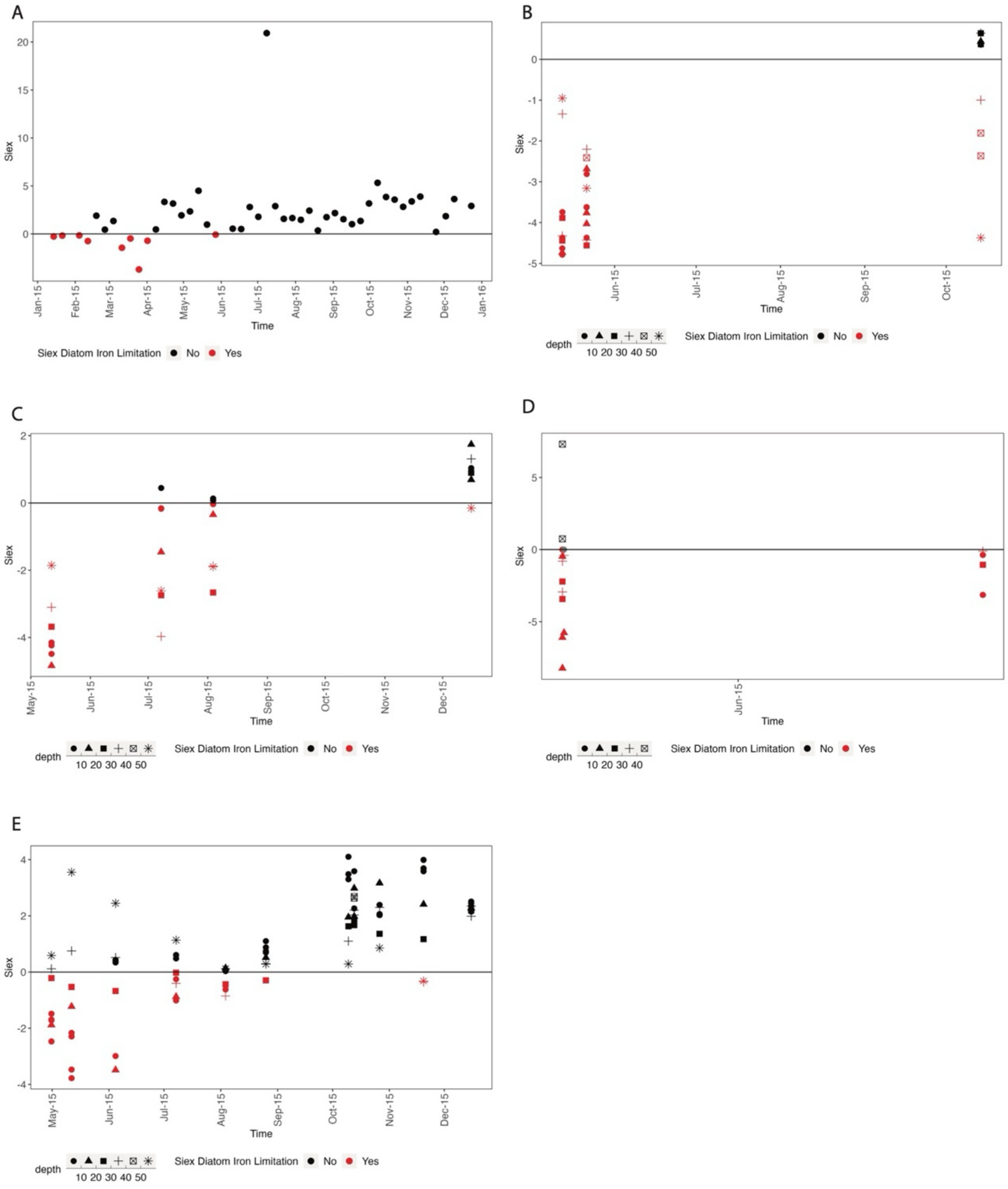

Figure S14. Calculations of Si_{ex}, a proxy for diatom iron limitation, suggest iron limitation early in the year in Monterey Bay, CA in several locations shown in Figure S1. Negative values of Si_{ex} indicate that diatoms preferentially take up H_4SiO_4 relative to NO_3^- due to iron deficiency, and more negative Si_{ex} values indicate a higher level of iron deficiency. All calculations assume the ratio of H_4SiO_4 to NO_3^- at the regional upwelling source depth "R-preformed" is equal to 1, and include measurements from depths above 65 meters. Si_{ex} is calculated at (A) MWII, (B) MARS, (C) M2, (D) ESP South, and (E) C1.

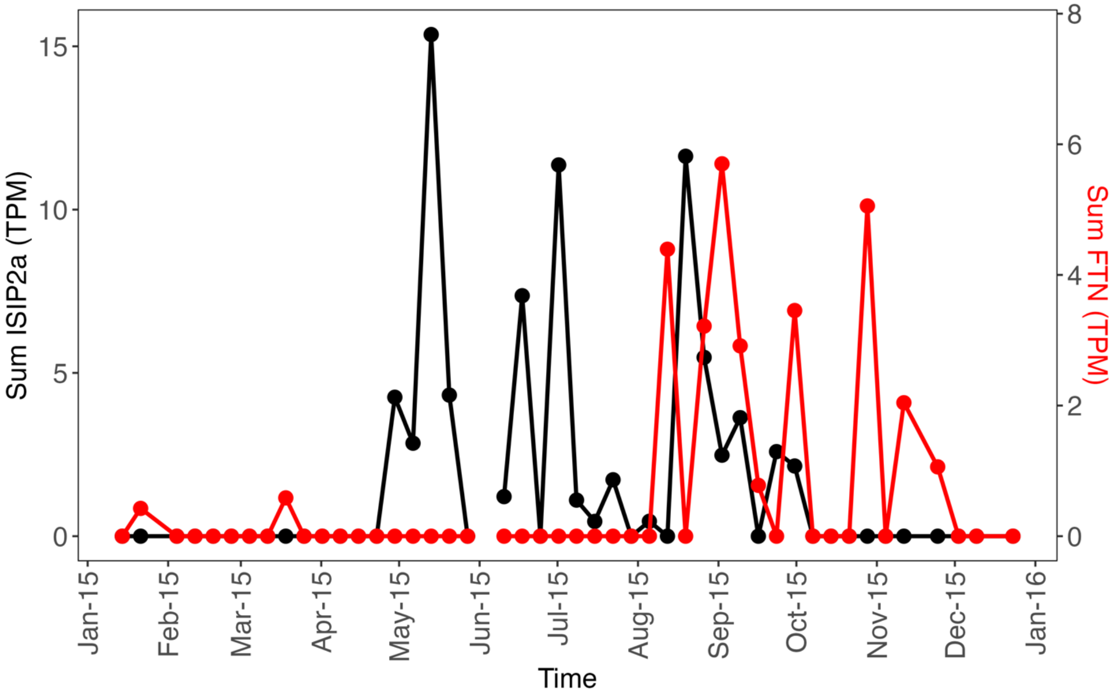

Figure S15. Sum of *Pseudo-nitzschia* expression of *ISIP2A* and ferritin (*FTN*) in transcripts per million (TPM).

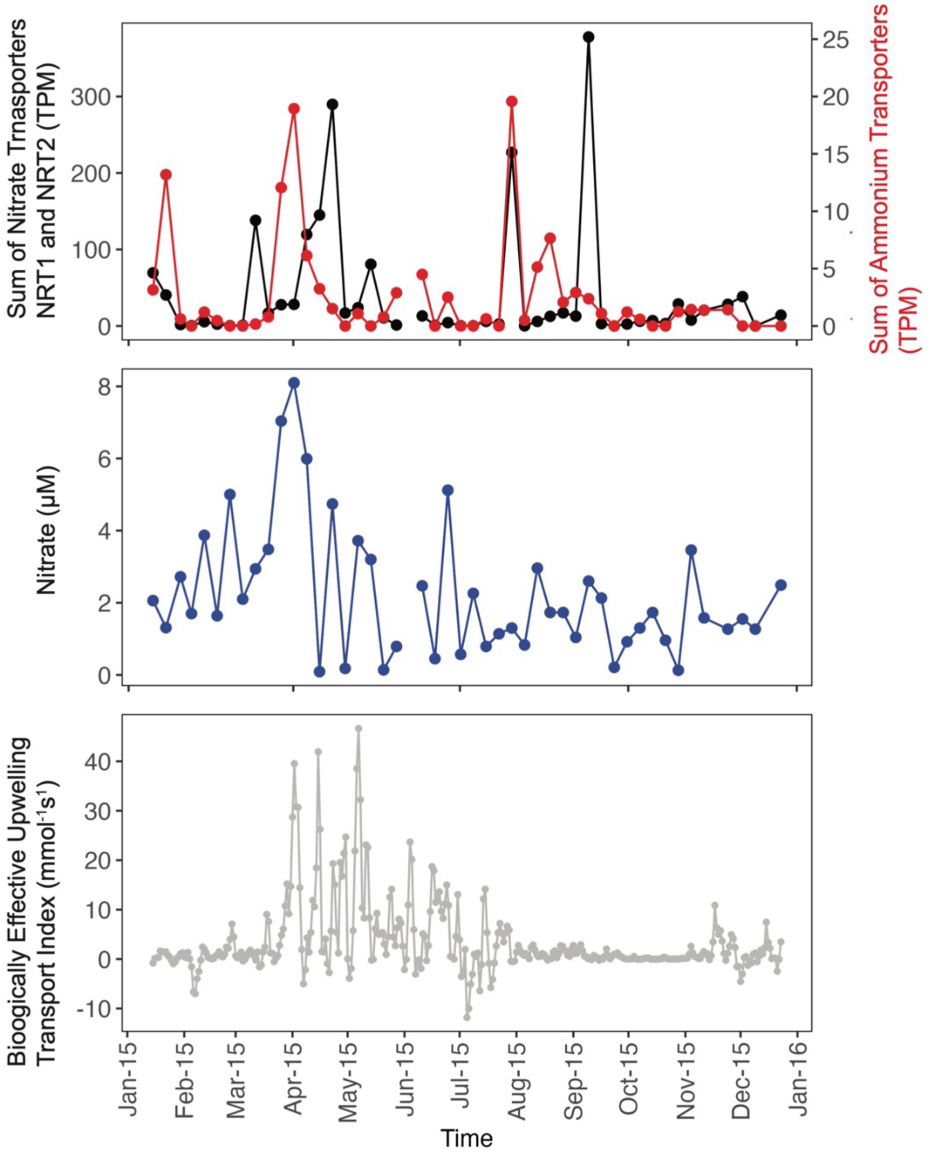

Figure S16. Diatom nitrogen transporters at Monterey Wharf peak in the Spring and late-Summer as a reflection of regional upwelling conditions. Reads for all transporters were summed and normalized per million library reads (TPM).

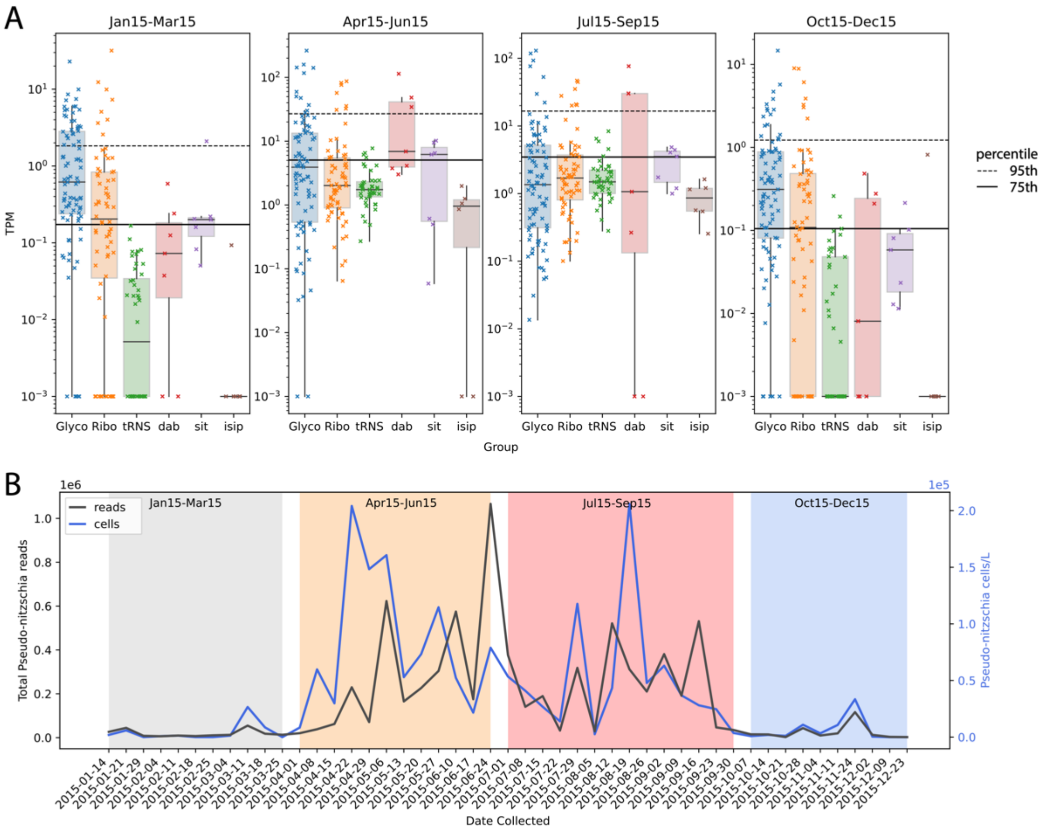

Figure S17. Function-wise gene expression in four periods of sampling. (*A*) Average transcription rates of three groups of housekeeping genes (glycolytic enzymes, ribosomal and translation proteins, tRNA-synthetases) were compared with the expression of *sit*, *dab*, and *isip2*/ferritin genes in four periods of sampling (January–March, April–June. July–September, October–December 2015). A pseudocount (10^{-3}) was added to all expression values. Many genes are detectable both in bloom and off-bloom seasons, while the number of undetected genes is low in bloom. Notably, target genes often showed comparable transcription rates as housekeeping genes. The data suggest sufficient sequencing coverage. (*B*) Total read counts and cell concentrations of *Pseudo-nitzschia* in the four periods above.

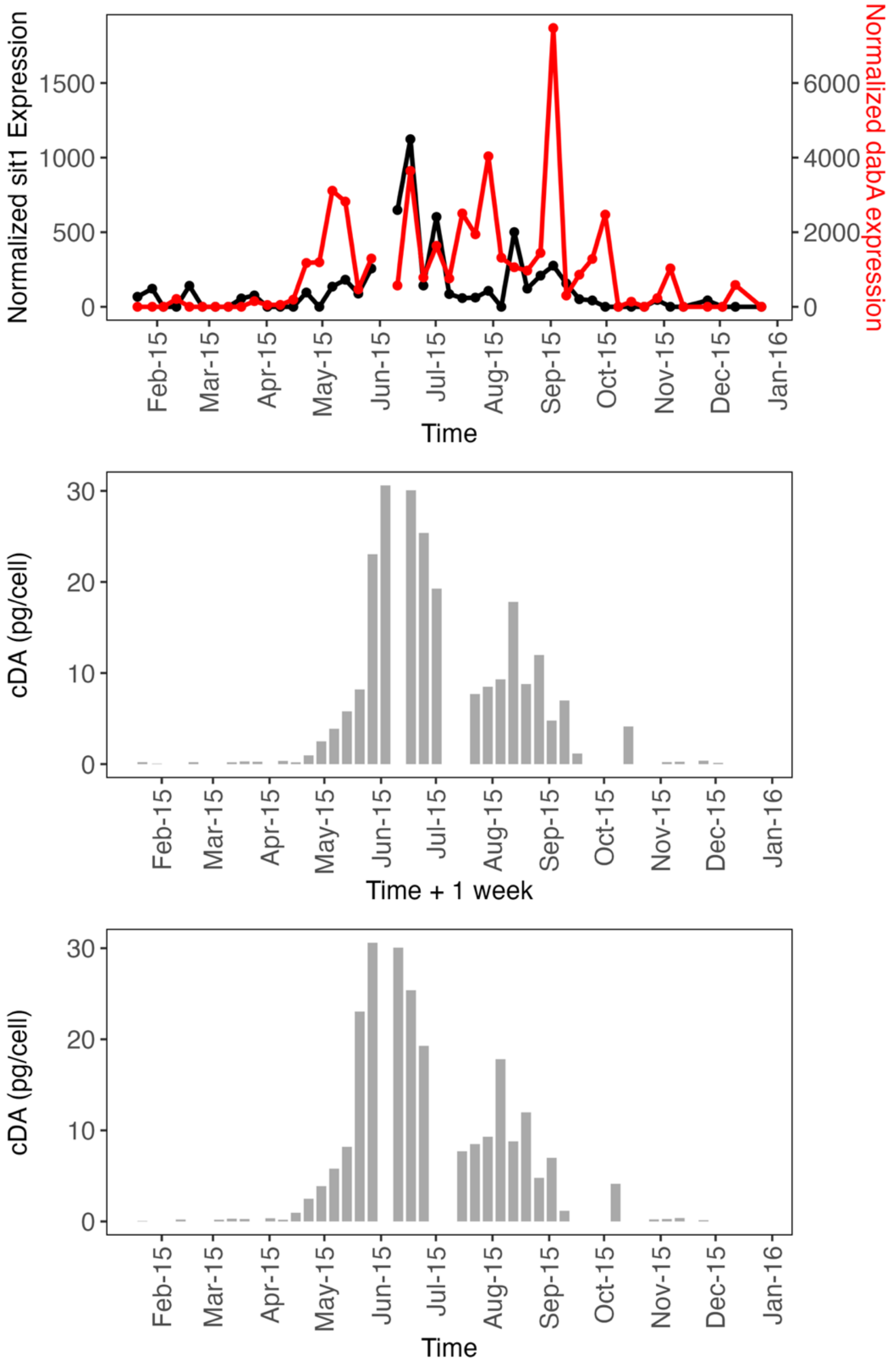

Figure S18. *Sit1* and *dabA* expression predict cDA in one week during the 2015 HAB. (Top) normalized *sit1* expression (black) and normalized *dabA* expression (red). Raw read counts of *sit1* and *dabA* from *Pseudo-nitzschia australis* were normalized to total *Pseudo-nitzschia* read counts and multiplied by 1.0 x 10^6. (Middle) cDA (pg/cell) offset by one week, and (bottom) cDA (pg/cell) concurrent in time with gene expression.

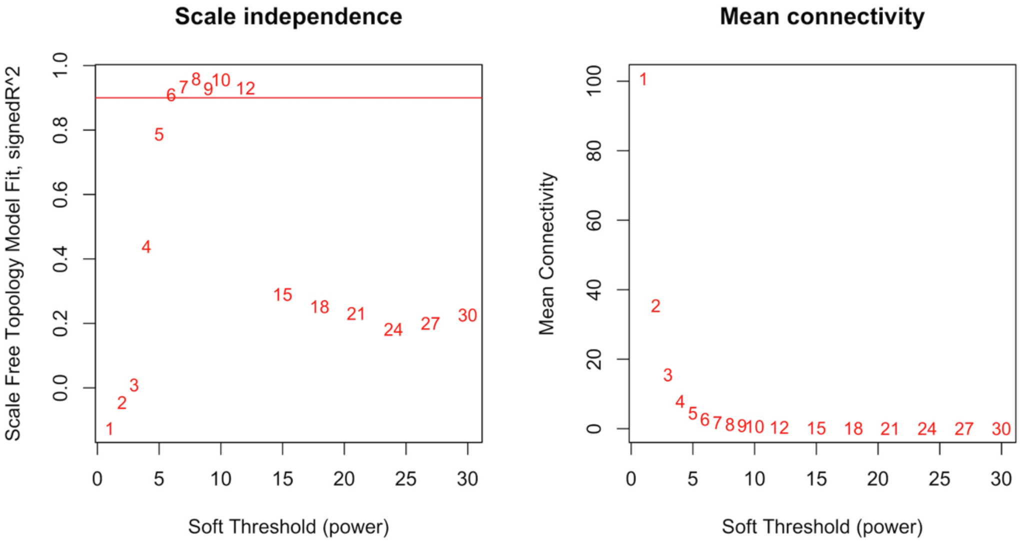

Figure S19. Determination of ideal soft-thresholding parameter “b” to test for the lowest value of “b” to exceed a scale-free topology R^2 value of 0.8, showing scale-free fit index and mean connectivity.

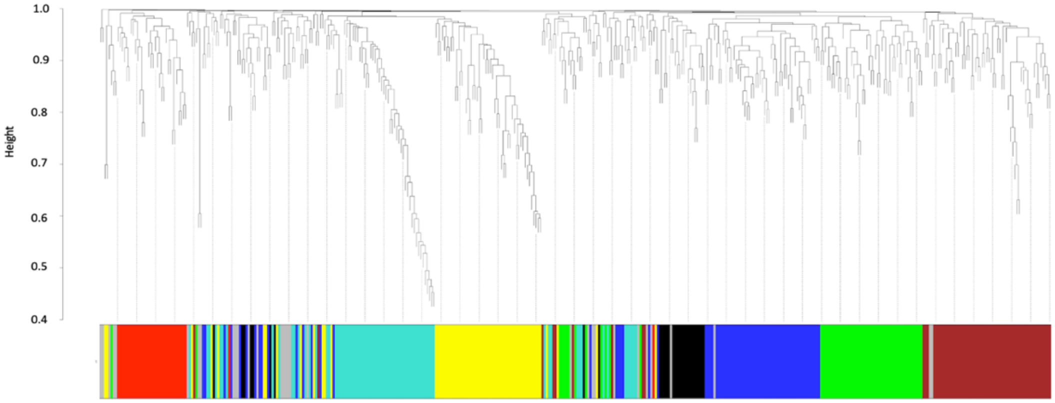

Figure S20. Clustering dendrogram of *P. australis* ORF expression profiles, together with assigned module colors.

Table S1. Sample metadata, including SRA accession numbers, collection dates, depth of sampling from plankton net tow, time of sampling, total raw paired reads, total trimmed reads, total mapped reads, and percentage of mapped reads. Additional counts for reads mapping to *Pseudo-nitzschia spp.* and *Pseudo-nitzschia australis* are also provided. All samples were taken from Monterey Wharf II (36.62 N - 121.89 W), collecting concentrated seawater from 5 meters depth by plankton net tow.

Sample name	SRA accession	Collection date	Time of Collection (PST)	Raw read pairs	Trimmed reads	Reads mapped	Reads mapped %	*P-n. spp.* reads	*P-n. australis* reads
112614_MWII	SRR26553412	11/26/2014	10:15	12453169	11346361	7442832	66%	6616	1554
120314_MWII	SRR26553410	12/3/2014	8:30	13673648	10670495	7266705	68%	9370	1099
121714_MWII	SRR26553408	12/17/2014	8:30	10466199	7289098	4589500	63%	3590	607
122314_MWII	SRR26553407	12/23/2014	9:41	9150459	1941745	892806	46%	2510	896
123014_MWII	SRR26553404	12/30/2014	10:15	11345692	6111852	3443320	56%	7077	1240
011415_MWII	SRR26553451	1/14/2015	8:00	15623471	7882435	4639906	59%	26562	3593
012115_MWII	SRR26553450	1/21/2015	9:40	15572224	6370578	3879865	61%	44572	6197
012915_MWII	SRR26553439	1/29/2015	815	12126783	8103586	4550149	56%	8233	1247
020415_MWII	SRR26553428	2/4/2015	8:15	8532571	7975861	4674890	59%	6312	1791
021115_MWII	SRR26553417	2/11/2015	8:30	4976482	3887137	2037037	52%	9545	2065
021815_MWII	SRR26553406	2/18/2015	8:20	13678504	8741526	4986971	57%	7113	882
022515_MWII	SRR26553403	2/25/2015	8:15	11039702	8915933	5804217	65%	10668	1342
030415_MWII	SRR26553402	3/4/2015	8:15	11191356	12698690	8441945	66%	12766	1231
031115_MWII	SRR26553401	3/11/2015	9:00	13937243	6896708	4334620	63%	54983	8274
031815_MWII	SRR26553400	3/18/2015	8:30	10637948	9107445	5879372	65%	17942	2551
032515_MWII	SRR26553449	3/25/2015	9:15	17044262	4518373	3109525	69%	13128	1862
040115_MWII	SRR26553448	4/1/2015	9:20	11569104	3869513	2846530	74%	20375	1979
040815_MWII	SRR26553447	4/8/2015	8:15	10812908	2868190	1997662	70%	38292	3974
041515_MWII	SRR26553446	4/15/2015	8:15	11046900	3852903	2415660	63%	62440	10116
042215_MWII	SRR26553445	4/22/2015	9:15	15979324	4647146	3103948	67%	229604	83016
042915_MWII	SRR26553444	4/29/2015	8:15	11832924	5701041	2666001	47%	71148	39068
050615_MWII	SRR26553443	5/6/2015	8:30	15651825	3414059	2294804	67%	622963	430202
051315_MWII	SRR26553442	5/13/2015	8:30	12866371	4605414	3098185	67%	165172	103808
052015_MWII	SRR26553441	5/20/2015	8:15	9426251	3521005	2285343	65%	226369	115843
052715_MWII	SRR26553440	5/27/2015	9:00	10326828	3725656	2605081	70%	304194	123265
061015_MWII	SRR26553438	6/10/2015	10:00	12823943	4932289	3187616	65%	575008	385774
061715_MWII	SRR26553437	6/17/2015	9:40	11337184	8130315	5172837	64%	175357	129740
062415_MWII	SRR26553436	6/24/2015	10:00	17490843	7043662	4816689	68%	1066184	787851
070115_MWII	SRR26553435	7/1/2015	10:45	14830412	8025450	5073430	63%	376673	275281
070815_MWII	SRR26553434	7/8/2015	7:45	17206426	10177238	6040383	59%	140753	103015
071515_MWII	SRR26553433	7/15/2015	8:15	17988320	10306203	5959134	58%	189408	142477
072215_MWII	SRR26553432	7/22/2015	9:30	17192893	10947290	6589011	60%	32409	21471
072915_MWII	SRR26553431	7/29/2015	9:30	7142975	5043703	2903914	58%	318145	252579
080515_MWII	SRR26553430	8/5/2015	9:00	9103091	11588896	7397204	64%	28865	13573

081215_MWII	SRR26553429	8/12/2015	8:15	5366862	6251397	3761394	60%	521256	420976
081915_MWII	SRR26553427	8/19/2015	8:15	11659645	1988772	1317879	66%	311993	203755
082615_MWII	SRR26553426	8/26/2015	11:15	12863640	4270693	2375112	56%	210372	121833
090215_MWII	SRR26553425	9/2/2015	8:15	17062229	20922652	11492819	55%	380793	289829
090915_MWII	SRR26553424	9/9/2015	8:30	16793064	27517467	18158283	66%	191490	54696
091615_MWII	SRR26553423	9/16/2015	8:30	10050361	15553559	10328443	66%	531002	422974
092315_MWII	SRR26553422	9/23/2015	8:30	9567311	12534840	8234921	66%	46758	31676
093015_MWII	SRR26553421	9/30/2015	8:15	8970710	14622469	9384255	64%	34807	16031
100715_MWII	SRR26553420	10/7/2015	8:30	9368978	11629762	7192978	62%	15112	1778
101415_MWII	SRR26553419	10/14/2015	8:10	8936304	9996850	5985296	60%	14610	2483
102115_MWII	SRR26553418	10/21/2015	8:15	6604356	5686156	3465651	61%	2682	533
102815_MWII	SRR26553416	10/28/2015	8:30	9697620	10567021	7075740	67%	43366	4677
110415_MWII	SRR26553415	11/4/2015	8:15	12790513	8151483	4748373	58%	9714	2619
111115_MWII	SRR26553414	11/11/2015	8:45	12909199	7536228	4858324	64%	19582	2007
112415_MWII	SRR26553413	11/24/2015	9:15	14794094	24681765	14506857	59%	116548	9939
120215_MWII	SRR26553411	12/2/2015	9:30	9305564	9959116	6488678	65%	12780	1714
120915_MWII	SRR26553409	12/9/2015	8:15	8166670	7650559	5118357	67%	3416	741
122315_MWII	SRR26553405	12/23/2015	9:00	6308688	7844972	4413061	56%	6279	1026

Table S2. Amino acid sequences of several *dab* transcripts in the polyA-enriched RNA-sequencing dataset compared to known *dab* genes from *P. australis, P. multiseries*, and *P. seriata*.

MWII RAP sequence ID	Sequence	Literature Sequence	Reference	BLAST pair-wise align percent ID
seriata_dabA_contig_519263_1_1377_-	MKFSASIIATLATTGAAFTATPQKAFTAGASTRQQVSLDALSTLSSIGSLTAESRSEIVARVKETGLTLTNPKDLYWMVDHFKENYYDEGNYFYPIKTVCDGESIDVKFYCPFEPSLSPHYLQLYGTRDERASIYDTTMAKYNKINSEKTSAICTPYSSFGDTQIIAYFYSMMYYINDQTAHLKLPEGEIEADLIDVLNDDILVYLNEFLSVFEPEDPADFERIWDFLEFYQPYFNKVDGKIVLDEKYNKSTPTQMPLIQTICSYIADNFAPNKNVTQVIWEVIRYIKGVKNEIQIRGDKGFTLSLQEYDDFRDKVTASPMAHAVSDLTHKRFTYEAYTDPLFMELENRCSEIITYFNDVCTSDRERLDDDPFNSVFILMDLDPTLNFAASCDLVVGHAYEKMERFLELKEEILVSARSEKEKLAYAQMIKTREDSLIGYVLHEVCCVEDGFARDHKPL	MKFSASIIATLATTGAAFTATPHKAFTAGASTRQQVSLDALSTLSSIGSLTAESRSEIVARVKETGLTLTNPKDLYWMVDHFKENYYDEGNYFYPIKTVCDGESIDVKFYCPFEPSLSPHYLQLYGTRDERASIYDTTMAKYNKINSEKTSAICTPYSSFGDTQIIAYFYSMMYYINDQTAHLKLPEGDIEADLIDVLNDDILVYLNEFLSVFEPEDPADFERIWDFLEFYQPYFNKVDGKIVLDEKYNKSIPTQMPLIKTICSYIADNFAPNKNVTQVIWEVIRYIKGVKNEIQIRGDKGFTLSLQEYDDFRDKVTASPMAHAVSDLTHKRFTYEAYTDPLFMELENRCSEIITYFNDVCTSDRERLDDDPFNSVFILMDLDPTLNFAASCDLVVGHAHEKMERFLELKEEILVSARSEKEKLAYAQMIKTREDSLIGYVLHEVCCVEDGFARDHKPLMKAFLEEELTEALEMS	Haroardóttir *et al.*, 2019 *BMC Molecular Biology*	98.9
australis_dabA_contig_469576_120_1547_-	MKFSATILATLATTGAAFTANPQKAFTAGASARQQVSLNALSTLSSIGSLTAESKSEILSRVKENGLTLTNPKDLYWMVDHFKENYYDEGDYYYPIKTVCDGESIDVKFYCPFEPSLSPHYLQLYGSRDERASIYETTMAKYNKINSEKTSAICTPYSSYGDTQIIAYFYSMMYYINDQTAHLKLPEGEIEAELIDVLNDDILIYLNEFLSVFEPEDDADFERIWDFLEFYQPYFNKVDGKIVLDEKYQKGTPSQMPLIQTICSYIAEQFAPNKNITQVIWEVIRYIKGVKNEIQIRGDKGFTLSLQEYDDFRDKVTASPMAHAVSDLTHKRFTYEAYTDPLFMELENRCSEIITYFNDVCTSDRERLDDDPFNSVFILMDLDPTLNFAASCDLVVGHAYEKMERFLELKEEILVSARSEKEKLAFAQMIKTREDSLIGYVLHEVCCVEDGYARDHKPLMKAFLEEELTEALEMA	TLATTGAAFTANPQKAFTAGASARQQVSLNALSTLSSIGSLTAESKSEILSRVKENGLTLTNPKDLYWMVDHFKENYYDEGDYYYPIKTVCDGESIDVKFYCPFEPSLSPHYLQLYGSRDERASIYETTMAKYNKINSEKTSAICTPYSSYGDTQIIAYFYSMMYYINDQTAHLKLPEGEIEAELIDVLNDDILIYLNEFLSVFEPEDDADFERIWDFLEFYQPYFNKVDGKIVLDEKYQKGTPSQMPLIQTICSYIAEQFAPNKNITQVIWEVIRYIKGVKNEIQIRGDKGFTLSLQEYDDFRDKVTASPMAHAVSDLTHKRFTYEAYTDPLFMELENRCSEIITYFNDVCTSDRERLDDDPFNSVFILMDLDPTLNFAASCDLVVGHAYEKMERFLELKEEILVSARSEKEKLAFAQMIKTREDSLIGYVLHEVCCVEDGYARDHKPLMKAFLEEELTEALEMA	Brunson & McKinnie *et al.,* 2018, *Science* (identified from transcriptomes generated in Keeling *et al.,* 2014 *PLOS Biology*)	100
multiseries_dabA_contig_392124_1_1473_+	MKFATSIVAAIATTGAAFTVIPQKLSHPSQLNALNTMGSISSITAESPKEVLSRVQDAGLTLTNPNDLYWMVDFLKEKYYDNGDYYYPIKTVCDGESIDVKFYCPFEPSLSPHYLELYGSRDERASIYETTMKKYNRINSEKTSAICTPYSSYGDTQIVAYFYSMMYYINDQTAHLKLPESEIESELIDILNDDILIYLNEFMSIFEPEDAQDLERIWDFLDFYQPYFSKVDDKIVLDEKYLVRTPSQMPLIKTICEYVSEQFAPSKNITQ	MKFATSIVAAIATTGAAFTVIPQKLSHPSQLNALNTMGSISSITAESPKEVLSRVQDAGLTLTNPNDLYWMVDFLKEKYYDNGDYYYPIKTVCDGESIDVKFYCPFEPSLSPHYLELYGSRDERASIYETTMKKYNRINSEKTSAICTPYSSYGDTQIVAYFYSMMYYINDQTAHLKLPESEIESELIDILNDDILIYLNEFMSIFEPEDAQDLERIWDFLDFYQPYFSKVDGKIVLDEKYLVRTPSQMPLIKTICEYVSEQFAPSKNITQVIWEVVRYIKGVKDEIHIRGDKSFTLSLQEYDDFRDKV	Brunson & McKinnie *et al.,* 2018, *Science*	99.79

	VIWEVVRYIKGVKDEIHIRGDKSFTLSLQEYDDFRDKVTASPMAHAVSDLTHERFSYEAYTNPAFMELENRCSEIITYFNDVCTSDRERLDEDPFNSVFILMDLDPSLNFAKSCDVVVEHAYNKMQAFLKLKEEILESASDEEERLALARMIKTREDSLIGYVLHEVCCVEDGYARDHKPLMKAFLEEEITKSLAEKVKFNPVESESVRLN	TASPMAHAVSDLTHERFSYEAYTNPAFMELENRCSEIITYFNDVCTSDRERLDEDPFNSVFILMDLDPSLNFAKSCDVVVEHAYNKMQAFLKLKEEILESASDEEERLALARMIKTREDSLIGYVLHEVCCVEDGYARDHKPLMKAFLEEEITKSLAEKVKFNPVESESVRLN		
multiseries_dabC_contig_392175_3_581_-	MTVAINNETVVLTPNEDDVQVNKGKTLETSFPPLKGDDLKWFPRSSLPAEIPAIDIGKVSTKEELEQFLVDIRKSGLFYIVNHGVPEEVSINVYNAFREFISTTTEEERMKYYTDTHFQNGGYVPFQGSSIRGGNLGKPQKDHVVKYFWRGPEVINRTPNEKFTEAHNMHHTETFKVAEKVIRTIFKALKLRF	MTVAINNETVVLTPNEDDVQVNKGKTLETSFPPLKGDDLKWFPRSSLPAEIPAIDIGKVSTKEELEQFLVDIRKSGLFYIVNHGVPEEVSINVYNAFREFISTTTEEERMKYYTDTHFQNGGYVPFQGSSIRGGNLGKPQKDHVVKYFWRGPEVINRTPNEKFTEAHNMHHTETFKVAEKVIRTIFKALKLRFPDFDPMEFENTINSKKMFFTNRIYPQAEPSDEEEITHRLVPHLDTSFITLANQVPADNGFQGLFVETGDGKKVKVPGIRNSYLVFIGQSLSYLTKNYLPSALHGVDKPPSDLFEGSERSSLITFYEPAEIIIPSKNINPNPEETSESCPFFYDIGLTVNDPEGTTWDFVKNKFITGYYAD*	Brunson & McKinnie *et al.*, 2018, *Science*	100
multiseries_dabC_contig_698932_2_493_-	DFDPMEFENTINSKKMFFTNRIYPQAEPSDEEEITHRLVPHLDTSFITLANQVPADNGFQGLFVETGDGKKVKVPGIRNSYLVFIGQSLSYLTKNYLPSALHGVDKPPSDLFEGSERSSLITFYEPAEIIIPSKNINPNPEETSESCPFFYDIGLTVNDPEGTT	MTVAINNETVVLTPNEDDVQVNKGKTLETSFPPLKGDDLKWFPRSSLPAEIPAIDIGKVSTKEELEQFLVDIRKSGLFYIVNHGVPEEVSINVYNAFREFISTTTEEERMKYYTDTHFQNGGYVPFQGSSIRGGNLGKPQKDHVVKYFWRGPEVINRTPNEKFTEAHNMHHTETFKVAEKVIRTIFKALKLRFPDFDPMEFENTINSKKMFFTNRIYPQAEPSDEEEITHRLVPHLDTSFITLANQVPADNGFQGLFVETGDGKKVKVPGIRNSYLVFIGQSLSYLTKNYLPSALHGVDKPPSDLFEGSERSSLITFYEPAEIIIPSKNINPNPEETSESCPFFYDIGLTVNDPEGTTWDFVKNKFITGYYAD*	Brunson & McKinnie *et al.*, 2018, *Science*	100
australis_dabC_contig_519989_125_1249_-	MTVQVINNETNVLTPNEDDVQINKGKTLETRFPPLKADDLKWFPHDSLPAEIPAIDISKVNTKEELEQFLVDIRKSGLFYIVNHGVPEEVSINVYNAFREFLSTTTEEERMKYYTDTHFQNGGYVPFQGSSIRGGNLGKPQKDHVIKYFWRGPQVVNRTPSESFTKAHDDHHTETFNVAEKVIRTIFKALKLRFPDFDPMEFENTINSKKMFFTNRIYPQAEKSDEEELTHRLVPHLDTSFITLANQVPADNRFQGLFVETGDGKKVPVPGIRNSYLVFIGQSLSFLTKNYLPSALHGVDKPPKEIFEGSERSSLITFYEPAEIIIPSKNINPNPDEVTVSCPFFYDSIGLNVNDPEGTTWDFVKNKFIKGYYAD	MTVQVINNETNVLTPNEDDVQINKGKTLETRFPPLKADDLKWFPHDSLPAEIPAIDISKVNTKEELEQFLVDIRKSGLFYIVNHGVPEEVSINVYNAFREFLSTTTEEERMKYYTDTHFQNGGYVPFQGSSIRGGNLGKPQKDHVIKYFWRGPQVVNRTPSESFTKAHDDHHTETFNVAEKVIRTIFKALKLRFPDFDPMEFENTINSKKMFFTNRIYPQAEKSDEEELTHRLVPHLDTSFITLANQVPADNRFQGLFVETGDGKKVPVPGIRNSYLVFIGQSLSFLTKNYLPSALHGVDKPPKEIFEGSERSSLITFYEPAEIIIPSKNINPNPDEVTVSCPFFYDSIGLNVNDPEGTTWDFVKNKFIKGYYAD	Brunson & McKinnie *et al.,* 2018, *Science* (identified from transcriptomes generated in Keeling *et al.,* 2014 *PLOS Biology*)	100

australis_dabD_contig_383882_249_1943_-	MSSINVVTALVSIGLAVSAKFASSYHDFLSSPKSDEVGFLETLDITNTTSIDAVAHVVLGLVSYFVLSFARSYYKFRFSPLRDAPGFGPRSFVYGMFYEFLEAPFMEPPILALKKLREGGKEIPFLAYTTLFGSQRLLLLDCDLIKHVFTAPSGKDPMRYPKHYVYLREVVGDGLVVVEGQEWSRHRRIIQPAFQSMFLKDAIGMVVPALVENLVNVWKKTAGTTINMNAHLSLITLDVIGKVAFSHEFNASKLLNQWAESPDKELGEVDDPLISSIGGSFSSSPLKLMLTVLKLPWLEKHLSPSFRTTRNLLNKAADDIVQNARNIKDPSRRSVLNLMMEAKDGESSKARNQLTDTELRDEVKTFLVAGHETTSTWSHWALYVLAIRPDLQEKVYADVMKHAPPNDETIVLEQADQMEYMWAFMNETLRLYSPLGLISRVTHQEENFKGYTIPKGTNLRIPIHLIHRHPDHWKDPEVFRPERWFDKEETSKRHKFAFIPFAAGGRNCIGQRFATMEAKIIVANVAKNFKIHLADSMKGKEITFSNFISLKCNPEVEIRVEARK	MSSINVVTALVSIGLAVSAKFASSYHDFLSSPKSDEVGFLETLDITNTTSIDAVAHVVLGLVSYFVLSFARSYYKFRFSPLRDAPGFGPRSFVYGMFYEFLEAPFMEPPILALKKLREGGKEIPFLAYTTLFGSQRLLLLDCDLIKHVFTAPSGKDPMRYPKHYVYLREVVGDGLVVVEGQEWSRHRRIIQPAFQSMFLKDAIGMVVPALVENLVNVWKKTAGTTINMNAHLSLITLDVIGKVAFSHEFNASKLLNQWAESPDKELGEVDDPLISSIGGSFSSSPLKLMLTVLKLPWLEKHLSPSFRTTRNLLNKAADDIVQNARNIKDPSRRSVLNLMMEAKDGESSKARNQLTDTELRDEVKTFLVAGHETTSTWSHWALYVLAIRPDLQEKVYADVMKHAPPNDETIVLEQADQMEYMWAFMNETLRLYSPLGLISRVTHQEENFKGYTIPKGTNLRIPIHLIHRHPDHWKDPEVFRPERWFDKEETSKRHKFAFIPFAAGGRNCIGQRFATMEAKIIVANVAKNFKIHLADSMKGKEITFSNFISLKCNPEVEIRVEARK	Brunson & McKinnie *et al.*, 2018, *Science* (identified from transcriptomes generated in Keeling *et al.*, 2014 *PLOS Biology*)	100

Table S3. Contigs encoding *dab* genes from *de novo* metatranscriptomic assembly.

orf_id	***dab*** **gene**	**Producing species**	**Dates Detected**
contig_392124_1_1473_+	*dabA*	*P. multiseries*	4/1 - 5/13
contig_469576_120_1547_-	*dabA*	*P. australis*	4/15 – 9/30
contig_519263_1_1377_-	*dabA*	*P. seriata*	4/22 – 6/10
contig_390219_292_1467_-	*dabC*	*unknown*	3/11-4/08
contig_392175_3_581_-<br>contig_698932_2_493_-	*dabC*	*P. multiseries*	4/1 – 5/20
contig_519989_125_1249_-	*dabC*	*P. australis*	4/15 – 9/30
contig_383882_249_1943_-	*dabD*	*P. australis*	4/15 – 9/30

Table S4. ORF IDs of *sit1* genes expressed by *Pseudo-nitzschia* by metatranscriptomic assembly and species identified by best blast hit. The two contigs with best hits to *P. australis* were used in the *dabA* predictive model.

**orf_id**	**gene**	**Species**
contig_394674_286_1935_-	*sit1*	*Pseudo-nitzschia australis*
contig_520682_183_1781_-	*sit1*	*Pseudo-nitzschia australis*
contig_204427_3_611_-	*sit1*	*Pseudo-nitzschia delicatissima*
contig_1214386_114_920_-	*sit1*	*Pseudo-nitzschia heimii*
contig_356337_228_1880_-	*sit1*	*Pseudo-nitzschia heimii*
contig_37454_67_897_+	*sit1*	*Pseudo-nitzschia multiseries*
contig_397601_164_1882_-	*sit1*	*Pseudo-nitzschia multiseries*

Table S5. ORF IDs of *ISIP* and *FTN* genes expressed by *Pseudo-nitzschia* by metatranscriptomic assembly and species identified by best blast hit.

**orf_id**	**gene**	**Species**
contig_396772_186_1550_-	*ISIP2A*; iron starvation induced protein	*Pseudo-nitzschia australis*
contig_706447_2_475_-	*ISIP2A*; iron starvation induced protein	*Pseudo-nitzschia australis*
contig_822451_757_1515_-	*ISIP2A*; iron starvation induced protein	*Pseudo-nitzschia australis*
contig_556678_55_1098_-	*ISIP2B*; iron starvation induced protein	*Pseudo-nitzschia australis*
contig_940239_2_565_-	*FTN*	*Pseudo-nitzschia heimii*
contig_265879_21_689_-	*FTN*	*Pseudo-nitzschia pungens*

Table S6. Statistical results from multivariable generalized linear models used to predict domoic acid. Independent variable gene expression was specifically from *Pseudo-nitzschia australis*, and all raw counts were normalized to total *Pseudo-nitzschia* read counts.

	**Time Frame**	**Gene (*P. australis* raw read counts)**	**Normalization**	**Dependent variable**	**Akaike information criterion (AIC)**	**Multiple R^2**	**Adjusted R^2**	**p-value**
a	April-Sept	*sit1* *dabA*	Total *Pseudo-nitzschia* read counts	DA (pg/cell) in 1 week	139.41	0.598	0.543	3.34e-4
b	April-Sept	*sit1* *dabA*	Total *Pseudo-nitzschia* read counts	DA (pg/cell)	153.6	0.329	0.255	2.73e-2

Table S7. Domoic acid measurements from within and nearby Monterey Bay in 2015 that exceed 25 nM.

PLATFORM	DATE	LAT	LON	DEPTH	pDA (ng/L)	dDA (ng/L)	dDA (nM)
R/V Martin	5/7/15	36.90	-121.94	0	3051.90	54234.16	174.32
R/V Martin	5/29/15	36.83	-121.85	0	240.83	21240.15	68.27
R/V Martin	5/19/15	36.64	-121.92	12	12191.64	11817.14	37.98
R/V Martin	5/19/15	36.64	-121.88	20	408.53	10607.32	34.09
R/V Martin	5/19/15	36.64	-121.88	40	93.63	10189.48	32.75
R/V Martin	5/26/15	36.74	-121.91	21	1088.85	8532.63	27.43
R/V Martin	5/29/15	36.83	-121.85	14	510.48	8149.67	26.19
R/V Carson	6/5/15	36.61	-122.01	9	4212.16	35151.18	112.98
R/V Carson	6/5/15	36.70	-121.89	0	6361.49	20999.23	67.50
R/V Carson	6/5/15	36.64	-121.88	0	4485.38	19881.90	63.90
R/V Carson	5/28/15	36.78	-121.87	0	2128.14	17143.37	55.10
R/V Carson	6/5/15	36.78	-121.87	0	9641.05	13487.89	43.35
R/V Carson	6/5/15	36.70	-121.89	7	8301.68	10321.89	33.18
R/V Carson	5/12/15	36.90	-121.93	0	807.04	10096.42	32.45
R/V Carson	5/12/15	36.90	-121.93	8	1245.01	9312.03	29.93
R/V Carson	5/28/15	36.78	-121.87	60	171.78	8901.58	28.61
Shimada	6/29/15	36.45	-122.14	3	353.92	20294.34	65.23
Shimada	6/30/15	36.70	-122.24	3	3901.48	20018.15	64.34
Shimada	6/28/15	36.20	-122.15	3	1361.42	11981.32	38.51
Shimada	6/30/15	36.70	-122.03	3	5040.44	10328.06	33.20
Shimada	6/29/15	36.45	-122.56	3	186.00	8682.57	27.91
Shimada	6/29/15	36.45	-122.34	3	223.31	8398.60	27.00
Santa Cruz Wharf	11/25/15	36.96	-122.02	Mix 0, 1.5, and 3 m: equal volumes	13.35	980599.22	3151.84
Santa Cruz Wharf	7/31/15	36.96	-122.02	Mix 0, 1.5, and 3 m: equal volumes	132.77	12427.17	39.94

Table S8. Primers used in this study. Sequence in red and green represent Illumina sequencing adaptors, black sequence represents library specific index sequences (8 nt), blue sequence represents amplicon specific annealing sequence.

Amplicon	Primer name	Primer Sequence
18SV4	V4F	AATGATACGGCGACCACCGAGATCTACACTCTTTCCCTACACGACGCTCTTCCGATCTNNNNXXXXXXXXCCAGCASCYGCGGTAATTCC
18SV4	V4RB	CAAGCAGAAGACGGCATACGAGATXXXXXXXXTGACTGGAGTTCAGACGTGTGCTCTTCCGATCTACTTTCGTTCTTGATYR
16S	515F-Y	AATGATACGGCGACCACCGAGATCTACACTCTTTCCCTACACGACGCTCTTCCGATCTNNNNXXXXXXXXGTGYCAGCMGCCGCGGTAA
16S	926R	CAAGCAGAAGACGGCATACGAGATXXXXXXXXGTGACTGGAGTTCAGACGTGTGCTCTTCCGATCTCCGYCAATTYMTTTRAGTTT
ITS2	5.8SF	AATGATACGGCGACCACCGAGATCTACACTCTTTCCCTACACGACGCTCTTCCGATCTNNNNXXXXXXXXTGCTTGTCTGAGTGTCTGTGGA
ITS2	28SR	CAAGCAGAAGACGGCATACGAGATXXXXXXXXGTGACTGGAGTTCAGACGTGTGCTCTTCCGATCTTATGCTTAAATTCAGCGGGT

Dataset S1 (separate file). *Pseudo-nitzschia* ORFs Dataset, including ORF IDs, comprehensive annotation, best hit species and per-library read counts.

SI References

1. S. Balzano, E. Abs, S. Leterme, Protist diversity along a salinity gradient in a coastal lagoon. *Aquat. Microb. Ecol.* **74**, 263–277 (2015).

2. A. E. Parada, D. M. Needham, J. A. Fuhrman, Every base matters: assessing small subunit rRNA primers for marine microbiomes with mock communities, time series and global field samples. *Environ. Microbiol.* **18**, 1403–1414 (2016).

3. E. Bolyen, *et al.*, Reproducible, interactive, scalable and extensible microbiome data science using QIIME 2. *Nat. Biotechnol.* **37**, 852–857 (2019).

4. B. J. Callahan, *et al.*, DADA2: High-resolution sample inference from Illumina amplicon data. *Nat. Methods* **13**, 581–583 (2016).

5. L. Guillou, *et al.*, The Protist Ribosomal Reference database (PR2): a catalog of unicellular eukaryote small sub-unit rRNA sequences with curated taxonomy. *Nucleic Acids Res.* **41**, D597-604 (2013).

6. P. Yilmaz, *et al.*, The SILVA and “All-species Living Tree Project (LTP)” taxonomic frameworks. *Nucleic Acids Res.* **42**, D643-648 (2014).

7. J. Decelle, *et al.*, PhytoREF: a reference database of the plastidial 16S rRNA gene of photosynthetic eukaryotes with curated taxonomy. *Mol. Ecol. Resour.* **15**, 1435–1445 (2015).

8. H. C. Lim, *et al.*, Phylogeny and species delineation in the marine diatom *Pseudo-nitzschia* (Bacillariophyta) using cox1, LSU, and ITS2 rRNA genes: A perspective in character evolution. *J. Phycol.* **54**, 234–248 (2018).

9. P. J. McMurdie, S. Holmes, phyloseq: An R Package for Reproducible Interactive Analysis and Graphics of Microbiome Census Data. *PLOS ONE* **8**, e61217 (2013).

10. E. Bertrand, *et al.*, Phytoplankton-Bacterial Interactions Mediate Micronutrient Colimitation at the Coastal Antarctic Sea Ice Edge. *Proc. Natl. Acad. Sci. U.S.A.* **112,** 9938-9943 (2015).

11. R. Schmieder, Y. W. Lim, R. Edwards, Identification and removal of ribosomal RNA sequences from metatranscriptomes. *Bioinformatics* **28**, 433–435 (2012).

12. M. Rho, H. Tang, Y. Ye, FragGeneScan: predicting genes in short and error-prone reads. *Nucleic Acids Res.* **38**, e191 (2010).

13. S. Podell, T. Gaasterland, DarkHorse: a method for genome-wide prediction of horizontal gene transfer. *Genome Biol.* **8**, R16 (2007).

14. H. Li, Aligning sequence reads, clone sequences and assembly contigs with BWA-MEM (2013) (July 31, 2023).

15. C. A. Durkin, J. A. Koester, S. J. Bender, E. V. Armbrust, The evolution of silicon transporters in diatoms. *J. Phycol.* **52**, 716–731 (2016).

16. R. C. Edgar, Muscle5: High-accuracy alignment ensembles enable unbiased assessments of sequence homology and phylogeny. *Nat. Commun.* **13**, 6968 (2022).

17. S. Capella-Gutiérrez, J. M. Silla-Martínez, T. Gabaldón, trimAl: a tool for automated alignment trimming in large-scale phylogenetic analyses. *Bioinformatics* **25**, 1972–1973 (2009).

18. B. Q. Minh, *et al.*, IQ-TREE 2: New Models and Efficient Methods for Phylogenetic Inference in the Genomic Era. *Mol. Biol. Evol.* **37**, 1530–1534 (2020).

19. P. Langfelder, S. Horvath, WGCNA: an R package for weighted correlation network analysis. *BMC Bioinformatics* **9**, 559 (2008).

20. P. J. Keeling, *et al.*, The Marine Microbial Eukaryote Transcriptome Sequencing Project (MMETSP): Illuminating the Functional Diversity of Eukaryotic Life in the Oceans through Transcriptome Sequencing. *PLOS Biol.* **12**, e1001889 (2014).

21. S. L. Hogle, *et al.*, Pervasive iron limitation at subsurface chlorophyll maxima of the California Current. *Proc. Natl. Acad. Sci. U.S.A.* **115**, 13300–13305 (2018).

22. A. Marchetti, *et al.*, Development of a molecular-based index for assessing iron status in bloom-forming pennate diatoms. *J. Phycol.* **53**, 820–832 (2017).
